# Can structure speak for understanding? A dual assessment of systems thinking for sustainability in preservice STEM teachers’ concept maps

**DOI:** 10.3389/fpsyg.2026.1846628

**Published:** 2026-07-15

**Authors:** Xiaolan Song, Xiangdong Wang, Xiuhua Liang, Jiaqi Li, Li Pan, Linhe Lu

**Affiliations:** 1 Faculty of Education, Northeast Normal University, Changchun, China; 2 School of Geographical Sciences, Northeast Normal University, Changchun, China; 3 The National Research Institute for Teaching Materials, Changchun, China; 4 Academy for Research in Teacher Education, Northeast Normal University, Changchun, China; 5 School of Biological Sciences, University of Oklahoma, Norman, OK, United States

**Keywords:** assessment, concept mapping, preservice teachers, stem education, structural-semantic mismatch, sustainability education, systems thinking

## Abstract

**Introduction:**

Structural indicators extracted from concept maps, such as node counts, connection density, and cycle counts, are often used as proxies for systems thinking. However, it remains unclear whether a structurally complex concept map actually reflects deeper semantic understanding of a sustainability system. This study developed the Structural–Semantic Dual Assessment (SSDA) framework as an exploratory tool to examine whether and how structural indicators correspond to semantic understanding.

**Methods:**

Forty-seven preservice STEM teachers with a geography background completed a concept-mapping task on the evolution of the Lop Nur human–land system. The SSDA framework assessed three dimensions of systems thinking: Network Structuring, Nonlinear Mechanism Deconstruction, and Spatiotemporal Reasoning. For each dimension, graph-theoretic structural indicators were extracted from the maps and compared with expert semantic ratings. We further used correlation analysis, principal component analysis-based structural–semantic profiling, representative case analysis, and a supplementary causal-chain completeness analysis.

**Results:**

Most participants identified system elements and annotated spatiotemporal information with reasonable consistency, but feedback mechanisms showed a different pattern. Some participants described feedback loops coherently in writing yet drew few or no closed cycles in their maps. The correspondence between structural indicators and semantic scores varied by dimension: structural indicators showed moderate correspondence with spatiotemporal reasoning but almost no correspondence with mechanistic understanding. Cross-classifying structural and semantic scores produced four learner profiles, revealing structural–semantic mismatches in both directions. The supplementary causal-chain completeness analysis further showed that valid mechanism chains depended on both structural support and link validity, especially for development–restoration tensions, feedback relations, and cross-spatiotemporal governance.

**Discussion:**

These findings indicate that structural indicators alone provide only a partial picture of systems thinking in concept maps. Structural, semantic, and causal-chain evidence are best interpreted side by side and dimension by dimension. As an exploratory framework, SSDA may support preservice STEM teacher education by helping participants develop professional judgment in evaluating systems thinking.

## 1 Introduction

Education lies at the heart of the United Nations 2030 Agenda for Sustainable Development. Target 4.7 specifically requires that all learners gain the knowledge and skills needed to advance sustainability (United Nations, 2015). In response, countries and international organizations have developed competence frameworks for sustainability, aiming to give educators clearer guidance. The European Commission’s GreenComp framework, which organizes sustainability competencies into four areas and twelve components, places systems thinking at the core of “Embracing complexity in sustainability”—the capacity to examine issues from multiple perspectives and to trace interactions across time, space, and contexts (European Commission and Joint Research Centre, 2022). The OECD’s PISA 2025 science framework introduces the concept of “Agency in the Anthropocene,” a central expectation of which is that students understand how human activities shape Earth’s systems and develop regenerative solutions through systems thinking and creative thinking (OECD, 2023a). Though these frameworks approach sustainability competencies from different angles, all of them reposition education for sustainable development around competence rather than knowledge transmission; systems thinking recurs across each as a key element of this shift (Wiek et al., 2011; Lozano et al., 2017). This convergence is particularly salient in STEM education, where understanding complex Earth systems, managing resources, and evaluating human-environment interactions are central learning objectives (York et al., 2019).

Sustainability issues are complex in ways that put particular demands on systems thinking. Climate change, ecological degradation, resource depletion—issues that sit at the intersection of natural science, technology, and social systems—never occur in isolation. They cut across social, environmental, and economic systems. Their causal links cross multiple domains; effects lag behind causes by varying degrees; and the feedback loops involved operate across spatial scales that compound rather than simplify the picture (Alberti et al., 2020; Lawrence et al., 2020; Fu, 2023; Coletta et al., 2024). Sustainability issues demand that learners trace how elements within a system relate to and act upon one another. Yet systems thinking is difficult to observe directly, and conventional tests and questionnaires have proven limited in capturing it (Nosek and Riskind, 2012; Carter et al., 2015; OECD, 2023b). Modeling tasks, situational assessments, and concept mapping offer other possible routes (Sweeney and Sterman, 2000; Davis et al., 2023; Krab-Hüsken et al., 2023), but whether they can reliably capture systems thinking remains unresolved.

These frameworks are translated into students’ learning experiences largely through teachers’ instructional decisions (Fischer et al., 2022; Letouzey-Pasquier et al., 2023; Daumiller et al., 2025). What problems a teacher poses, which connections across natural and social systems get foregrounded, what kind of inquiry into real-world sustainability challenges students are asked to carry out—these choices shape whether and how learners engage with systems thinking in STEM-related contexts (Demssie et al., 2023; Li et al., 2023). From this perspective, preservice teachers’ understanding is also crucial in shaping the thinking patterns of their future students (Brandt et al., 2021; Kadji-Beltrán, 2024). Yet integrating complex issues and tracing relationships among system elements often proves difficult for preservice teachers (Kurent and Avsec, 2024). Assessment of preservice teachers’ systems thinking in sustainability-oriented STEM disciplines also remains limited—methods are fragmented, often relying on self-reports, and few tools exist that can reveal the deeper structure of thinking, particularly for disciplines that sit at the intersection of natural science and human-environment systems (Fischer et al., 2022; Vidal and Kuckuck, 2025).

Concept mapping has become a widely adopted tool for making learners’ knowledge structures visible in sustainability education across STEM disciplines, from chemistry and biology to geography and environmental science (Petrun Sayers et al., 2021; Krab-Hüsken et al., 2023; Liu and Liu, 2025). Researchers have taken different approaches to analyzing concept maps. Qualitative rubrics can reveal causal chains and feedback loops (Besterfield-Sacre et al., 2004; Szozda et al., 2023), yet the process is labor-intensive and does not scale easily (Watson et al., 2016; Bhatia et al., 2021). Automated network metrics are faster, but tend to conflate structural density with understanding, obscuring issues such as concept clustering, shallow reasoning, or missing intermediate mechanisms in causal chains (Deshpande and Ahmed, 2019; Bhatia et al., 2021). These two methods are seldom used in combination (Kinchin et al., 2019). Concept-map research has long distinguished relational, structural, and semantic levels of graphical analysis (Ifenthaler, 2010). In this study, each approach captures a different layer of evidence—structural metrics describe how concepts are organized, while qualitative judgments evaluate the validity and explanatory coherence of the represented relationships—and how these two layers correspond to each other has rarely been examined directly. In other words, structural indicators may reflect how learners externalize system elements and relations, but they do not by themselves show whether the represented links are valid or whether a causal chain is mechanistically complete. This raises a pressing question for STEM sustainability education: does a structurally complex concept map actually reflect deep understanding of the system, or does it merely signal information coverage?

To address this question, we develop the Structural–Semantic Dual Assessment (SSDA) framework, which collects structural and semantic evidence in parallel for each dimension of systems thinking and treats their alignment—or divergence—as the central analytic focus. Participants are preservice teachers majoring in geography—a discipline that occupies a distinctive position within STEM education by integrating natural science, social systems, and spatial analysis to address human-environment interactions across scales (Cox et al., 2019; Liu, 2023; Zhao et al., 2025). This disciplinary grounding makes geography a productive site for examining how STEM-oriented preservice teachers develop systems thinking in sustainability contexts (York et al., 2019). The task centers on “the evolution of the Lop Nur human–land system,” asking participants to map historical change, resource extraction, and ecological restoration in the region. In this case, changes in water resources, ecological degradation, potash extraction, and economic development pressures interact across natural and engineered systems in ways characteristic of the complex sustainability problems that STEM education is increasingly called upon to address. Building on this design, we ask three questions:

- RQ1: What patterns do preservice teachers’ concept maps show when examined separately along structural and semantic dimensions of the SSDA framework?
- RQ2: How does structural complexity relate to semantic quality in concept maps, and can structural indicators capture how deeply learners understand sustainability systems?
- RQ3: What distinct types of systems thinking can be identified through structural and semantic information, and what do these differences suggest for preparing STEM teachers in sustainability education?

## 2 Theoretical framework

Although both GreenComp and PISA 2025 treat systems thinking as central to sustainability competencies and outline its components in some detail (European Commission and Joint Research Centre, 2022; OECD, 2023a), neither offers concrete guidance on how this competency should be assessed. The SSDA framework proposed here is one attempt to fill that operational gap. Discipline-specific frameworks have also elaborated systems thinking as a way to connect components, interactions, and system-level behavior in STEM learning contexts (Momsen et al., 2022).

Drawing on these frameworks, the SSDA framework organizes systems thinking into three dimensions: Network Structuring (Dimension A), Nonlinear Mechanism Deconstruction (Dimension B), and Spatiotemporal Reasoning (Dimension C). For each dimension, the framework provides both structural and semantic analysis pathways, allowing the two to be compared. Network Structuring concerns whether learners can identify system elements and establish connections, while Nonlinear Mechanism Deconstruction examines their grasp of feedback loops and nonlinear dynamics. Spatiotemporal Reasoning (Dimension C) examines whether learners can establish causal links across temporal and spatial scales.

Table 1 summarizes how the three dimensions introduced above align with systems thinking competencies in GreenComp and PISA 2025.

**Table 1:** Alignment of SSDA dimensions with international frameworks.

SSDA dimension	Focus	GreenComp competency	PISA 2025 competency
Dimension A: network structuring	Identifying elements, building connections	Examining issues from multiple perspectives	Identifying and describing system elements
Dimension B: nonlinear mechanism deconstruction	Feedback loops, nonlinear dynamics	Understanding feedback loops and emergence	Analyzing interactions and dynamics
Dimension C: spatiotemporal reasoning	Cross-scale causal tracing	Understanding interactions across time, space, and context	Predicting and evaluating system behavior; cross-scale thinking

All three dimensions can be examined through concept maps, but this requires evidence from both structural and semantic levels. Structural indicators show how many concepts learners include, how connections are organized, and whether cycles or cross-scale links appear, but they cannot tell us whether these connections are accurate or the reasoning sound. Semantic analysis focuses on what learners label and the logic of their explanations, but without structural information, it may miss how learners organize knowledge as a whole.

**Figure 1:**
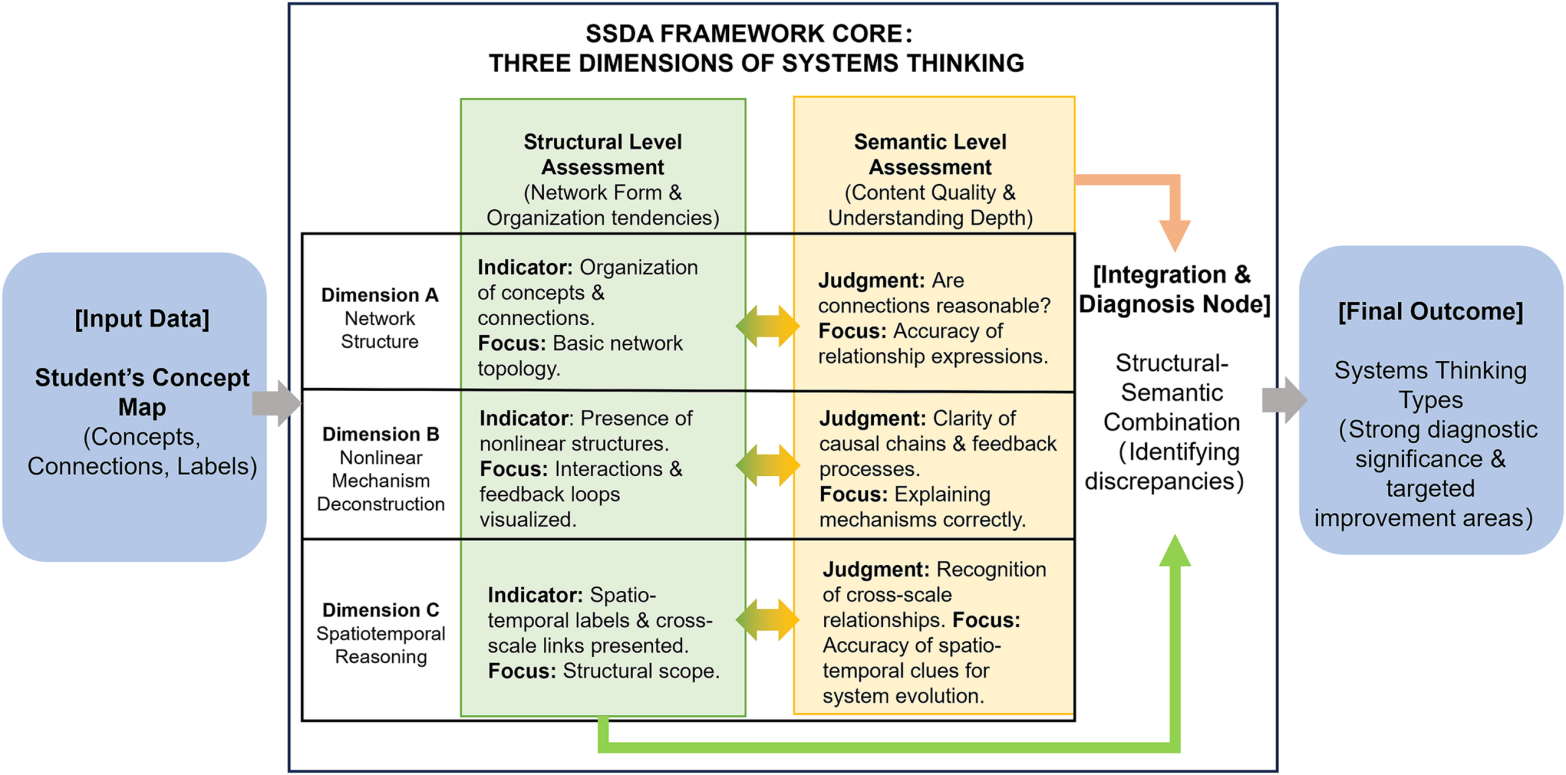
The Structural–Semantic Dual Assessment (SSDA) framework. A student’s concept map serves as input and is evaluated along two parallel tracks: Structural Level Assessment (green panel) and Semantic Level Assessment (yellow panel), each covering three dimensions—Network Structuring (Dimension A), Nonlinear Mechanism Deconstruction (Dimension B), and Spatiotemporal Reasoning (Dimension C). Bidirectional arrows indicate paired comparison between structural indicators and semantic judgments at each dimension. The converging green and orange arrows indicate that the final classification of systems thinking types is jointly determined by both sources of evidence.

The SSDA framework (Figure 1) addresses this by collecting both structural and semantic evidence for each dimension, enabling the two to be compared. Structural evidence captures the external organization of the map, whereas semantic evidence captures the interpreted validity and explanatory quality of the represented relationships. Convergence between the two increases confidence in the assessment judgment; divergence indicates that map structure and explanatory quality should be interpreted separately. For example, a learner may draw cyclic paths in a concept map but provide limited explanation of the underlying mechanism. Another learner may describe feedback mechanisms clearly in writing but fail to represent them as closed loops in the map. Reading both sources of evidence together therefore allows assessors to make a more cautious and dimension-specific judgment.

### 2.1 From components to networks: the structural basis of systems thinking

The Systems Thinking Hierarchy (STH) model describes this progression: learners typically begin by identifying system elements and establishing basic relationships before moving on to more complex mechanisms (Assaraf and Orion, 2010). Expert-novice research confirms this developmental pattern (Chi et al., 1981; Persky and Robinson, 2017). Organizing scattered elements into a coherent network is a key marker of systems thinking development (Assaraf and Orion, 2005; Bedinger et al., 2020; Mambrey et al., 2020).

Concept mapping makes this process visible (Kinchin et al., 2019; Eshuis et al., 2022). As learners draw, they turn their understanding of system elements into nodes and link them to show how these elements relate. The arrangement of nodes and links reveals how learners organize their understanding of the system (Evans and Jeong, 2023). The accuracy of links and relationship labels, in turn, reveals the quality of understanding (Srivastava et al., 2021).

Network Structuring thus forms Dimension A of the framework, with attention to whether learners move beyond isolated elements toward concept networks with discernible organizational logic.

### 2.2 From static structure to dynamic mechanisms: understanding nonlinearity

Changes in sustainability systems rarely unfold in a linear fashion. Ecological degradation, resource depletion, and similar problems resist prediction and management largely because they are shaped by feedback loops, time delays, and interactions that cut across scales. When these processes overlap, the trajectory of system change becomes far more complex than any unidirectional causal account would suggest (Likhoshvai et al., 2020; Wellmanns and Schmiemann, 2022; Procopio et al., 2025).

The Structure–Behavior–Function (SBF) framework offers one way to analyze these internal processes (Gero and Kannengiesser, 2004; Hmelo-Silver et al., 2007). “Behavior,” in SBF terms, denotes the changes that arise when system structures operate: the unfolding of causal chains, the transmission of information within the system, and the interactions among components (Hmelo-Silver, 2004; Hmelo-Silver et al., 2015). As nonlinear features, feedback loops and time delays belong to this behavioral level.

Concept maps can capture some of these features. Cyclic paths in a map may indicate an attempt to represent feedback, and bidirectional links may suggest recognition of reciprocal interactions among elements. Relationship labels add a layer that arrows cannot. A label distinguishing positive from negative feedback, for instance, reflects mechanistic reasoning at a level that no structural feature of the map can capture on its own (Schaffernicht, 2010; Neuwirth, 2020).

On this basis, the framework designates Nonlinear Mechanism Deconstruction as Dimension B, focusing on whether learners can identify and represent mechanisms that move beyond unidirectional linear causation.

### 2.3 Spatiotemporal coupling: reasoning across scales

In complex systems, important causal processes often unfold across different temporal and spatial scales (Wang, 2016; O’Donncha et al., 2022). Regional ecosystem degradation, for instance, may result from short-term changes in local water use, or from long-term climate shifts and large-scale land use changes. Grotzer and Solis identify “spatiotemporal disjunction” as a key barrier to understanding complex systems (Grotzer and Solis, 2015): learners struggle to link events that are distant in time or space yet causally connected (Davis, 2003; Khine and Saleh, 2013).

Geography’s sustained attention to scale offers a useful reference here (Ruddell and Wentz, 2009; Oshan et al., 2022): spatial nesting and temporal lag effects together shape how systems evolve (Bennett, 1975), and connecting across these scales often marks the boundary between surface and deeper understanding (Walker et al., 2017; Wu et al., 2019).

Concept maps offer two types of evidence for examining spatiotemporal reasoning. The first concerns whether learners add temporal or spatial labels to nodes and connect elements across scales. The second concerns whether these labels and connections are substantively sound and capture key turning points in system evolution.

Accordingly, this study defines Spatiotemporal Reasoning as Dimension C, examining whether learners can move beyond a single-scale view and integrate processes across different scales into a coherent causal context.

## 3 Materials and methods

### 3.1 Study design overview

**Figure 2:**
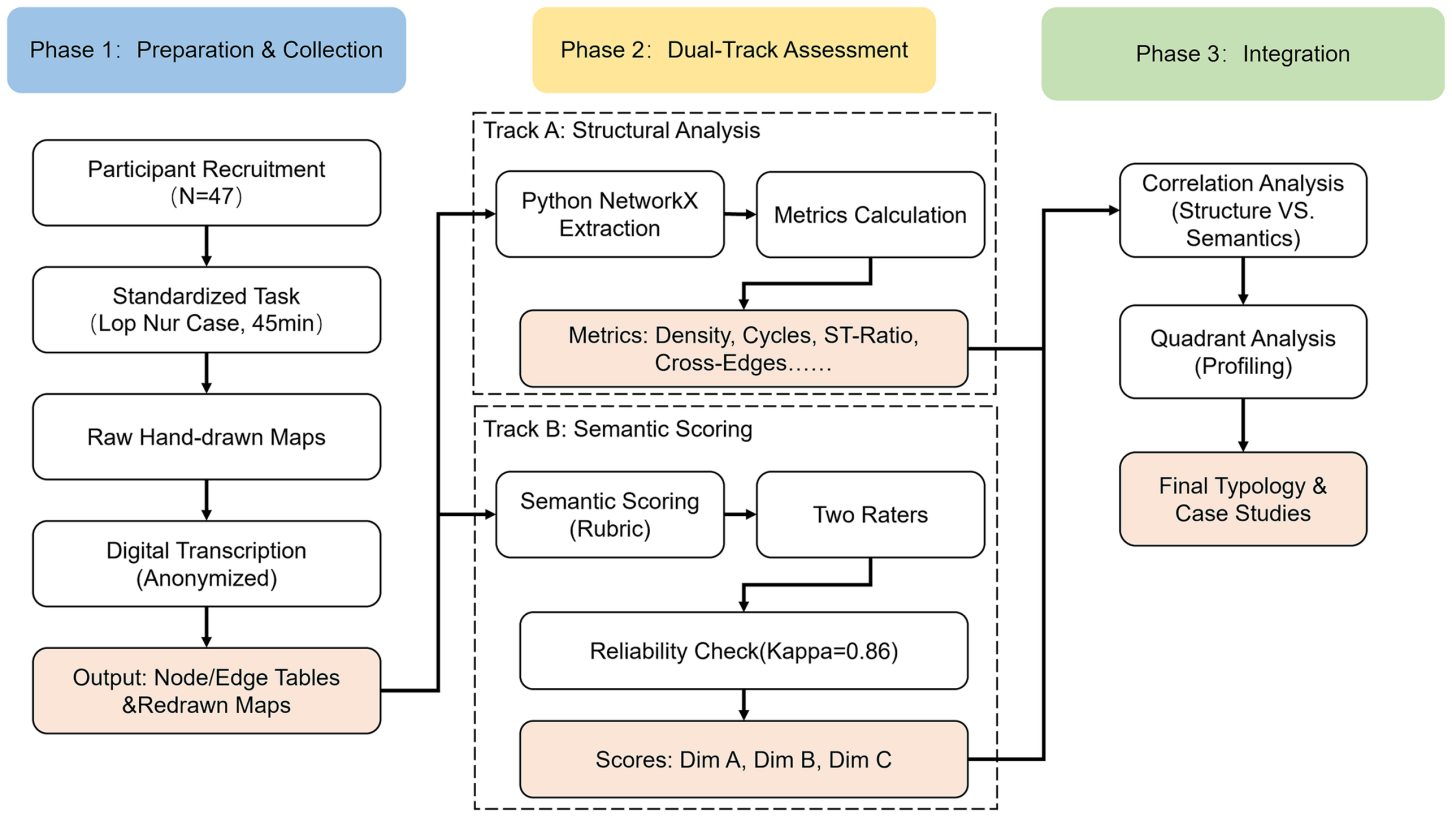
Overall methodological workflow of the study. The study proceeded in three phases. Phase 1 (Preparation and Collection) involved recruiting 47 preservice teachers majoring in geography, administering a standardized 45-min concept mapping task based on the Lop Nur human–land system case, and transcribing hand-drawn maps into anonymized node and edge tables. Phase 2 (Dual-Track Assessment) ran structural metric extraction via Python NetworkX and expert semantic scoring in parallel, with inter-rater reliability confirmed at *κ* = 0.86. Phase 3 (Integration) combined both outputs for correlation analysis, quadrant-based profiling, and representative case studies. ST: spatiotemporal; Dim A/B/C: Dimensions A, B, and C of the SSDA framework.

The study proceeded in three phases, outlined in Figure 2. The process began with participants completing a concept mapping task built around a sustainability scenario—the evolution of the Lop Nur human–land system—after which all hand-drawn maps were collected and organized as raw data for analysis. From each concept map, structural indicators and semantic scores were then generated separately, guided by the dimensions of the SSDA framework. The two sets of results were then brought together for correlation analysis and typological classification. Representative cases were selected to illustrate how structural and semantic evidence converged or diverged in participants’ concept maps.

### 3.2 Participants

The participants were 47 preservice teachers majoring in geography, recruited from a “Double First-Class” normal university in China, including 35 females and 12 males. All participants had completed their core coursework in geography by the time of data collection, though none had received dedicated training in systems thinking or system-oriented concept mapping.

Geography, as a STEM-related discipline, addresses research objects characterized by multi-factor interaction and multi-scale coupling (Zhao et al., 2016), making it a suitable context for studying how learners reason about complex sustainability systems. Such complex system contexts, including climate change, resource management, and ecological degradation, connect directly to sustainability issues and provide opportunities for participants to reason about systems thinking in a relatively authentic way. Geography teachers, moreover, often carry responsibility for sustainability-related topics across various curricula (Baerwald, 2010). In STEM education particularly, teachers serve as the primary conduit through which sustainability competence frameworks reach students, and preservice teachers’ grasp of systems thinking will shape both their future instructional choices and their students’ capacity to engage with complex systems. Assessing where preservice STEM teachers stand thus carries direct implications for sustainability education at scale.

This study was approved by the Institutional Review Board of Northeast Normal University (approval obtained prior to data collection). All participants provided written informed consent before participation and were informed of their right to withdraw at any stage without consequence. The collected concept maps were anonymized using participant codes and used exclusively for the purposes of this study.

### 3.3 Concept-mapping task

The concept-mapping task took “the evolution of the Lop Nur human–land system” as its topic. Lop Nur was selected because the case involves interlinked processes—changes in water resources, ecological degradation, potash mining—and economic development needs and ecological restoration goals compete with each other in ways that are recognizably similar to sustainability problems elsewhere. The task materials described the historical evolution of Lop Nur, resource extraction, and ecological management efforts in the region, and were accompanied by scaffolding prompts. Participants then drew concept maps showing how they understood the elements within the system and the way those elements connected.

Drawing took place in a single 45-min classroom session, the same for all participants. Participants identified key concepts from the materials and connected them with labeled links to indicate interactions. Participants were asked to add temporal or spatial annotations to nodes where applicable. Once the drawing was complete, participants went back over their maps. They were asked to mark any feedback loops and cross-spatiotemporal connections they could identify, and to provide written explanations where such explanations were included in the supplementary space. This additional step gave participants’ reasoning about feedback mechanisms and cross-scale relationships a separate outlet. Participants’ written descriptions of feedback loops and cross-scale relationships were used as supplementary evidence in semantic scoring and case interpretation, especially for judging whether they could explain feedback mechanisms and cross-spatiotemporal relations. These written records were not treated as complete representations of participants’ knowledge; rather, they were interpreted together with the nodes, directed links, relation labels, and annotations shown in the concept maps. Appendix A contains the full task instructions and the illustrative example used to explain cross-spatiotemporal links.

### 3.4 Data preparation and transcription

Once collected, the hand-drawn maps were coded (S01–S47) for anonymity. The first author then scanned each map into a digital file so that raters could score them independently. From these digital files, each map was transcribed into two components: a node table and an edge table. The node table contained concept names together with their temporal and spatial annotations. The edge table logged each link by source node, target node, and relationship label. Network structural indicators were computed from these two tables. The digital records and transcription preserved the original layout and annotations of each map as closely as possible. All transcription was completed by the first author. A second researcher independently checked a random sample of approximately 30% of the maps. Discrepancies were resolved through discussion.

### 3.5 Structural metrics

Each concept map was converted into a directed network, and structural features were computed using Python’s NetworkX library.

The network indicators were organized into groups corresponding to the three systems thinking dimensions defined in the SSDA framework (Table 2). Node and edge counts captured the overall scale of each concept map. Network density, average degree, and graph diameter were assigned to the “Network Structuring” dimension, characterizing both the interconnectedness and structural span of the concept network. Whether feedback or interaction structures appeared in the maps was captured by the number of cycles and reciprocal edges, corresponding to the “Nonlinear Mechanism Deconstruction” dimension. The “Spatiotemporal Reasoning” dimension was represented by the proportion of spatiotemporal labels and the number of cross-spatiotemporal links, which together reflected how much attention participants gave to system dynamics and coupling across scales.

**Table 2:** Structural indicators and their correspondence to the SSDA framework.

SSDA dimension	Indicator	Analytical meaning	Why it reflects this dimension
Overall scale	Number of nodes	Range of concepts included	More nodes indicate broader conceptual coverage
	Number of edges	Extent of explicit relations	More edges suggest richer expression of connections
Dimension A: network structuring	Network density	Degree of overall interconnectedness	Captures how tightly participants organize ideas
	Average degree	Typical number of connections per node	Indicates whether concepts are integrated or isolated
	Graph diameter	Longest conceptual distance	Reflects the overall structure and hierarchy of the network
Dimension B: nonlinear mechanism deconstruction	Number of cycles	Presence of feedback-like structures	Cycles represent potential causal loops
	Reciprocal edges	Bidirectional relations	Suggest recognition of two-way or reinforcing interactions
Dimension C: spatiotemporal reasoning	Proportion of spatiotemporal labels	Use of time/space information	Shows attention to system dynamics and scale
	Number of cross-spatiotemporal links	Connections across spatial or temporal levels	Indicates awareness of interactions across scales

### 3.6 Semantic scoring and reliability

The semantic scoring of SSDA uses a 0–3 scale for each of its three dimensions. It primarily evaluates two aspects: the quality of causal reasoning and the clarity with which the learner represents system mechanisms. Dimension A (Network Structuring) focuses on how the learner organizes concepts, assessing the quality of the conceptual network they construct. Dimension B (Nonlinear Mechanism Deconstruction) examines mechanistic reasoning by considering the feedback loops and bidirectional interactions the learner presents. Dimension C (Spatiotemporal Reasoning) reflects the learner’s presentation of spatiotemporal information, emphasizing whether it illustrates causal connections across time and space. The complete scoring rubric is provided in Appendix B. In this study, the distinction between structural and semantic analysis is analytical rather than absolute. Structural metrics were calculated from the graph form of the maps, treating nodes and directed edges as network units without judging the substantive validity of the concepts or links. Semantic scoring, by contrast, involved expert interpretation of the meanings of nodes, relation labels, causal directions, and the coherence of the represented mechanisms.

Scoring was performed by two raters: the first author, with a background in curriculum and instruction, and a professor in geography education. Prior to formal scoring, the two raters calibrated their judgments by jointly scoring five randomly selected concept maps, after which each independently scored the full set of samples.

Inter-rater reliability was examined with quadratic weighted Cohen’s kappa, a measure suited to ordinal rating data (de Raadt et al., 2021). The quadratic weighted Cohen’s kappa for the total semantic score was 0.86. The values for the three dimensions were 0.89 (A), 0.76 (B), and 0.62 (C). Dimension C yielded a lower kappa, most likely because scores clustered in the high range (M = 2.66, Mdn = 3), leaving little room for variation. Under such conditions, small scoring differences between raters produce disproportionately low kappa values (Feinstein and Cicchetti, 1990). The kappa for Dimension B (0.76) reflects the inherent complexity of judging mechanistic reasoning, where raters must assess not only whether feedback loops are identified but also the logical coherence of the causal connections described. Calibration discussion prior to independent scoring helped to align rater judgments on this dimension. For all subsequent analyses, each participant’s score was the mean of the two raters’ ratings.

### 3.7 Integrated structural-semantic analysis

After the structural indicators and semantic scores had been generated separately, the two sets of results were brought together for an integrated analysis. Spearman’s rank correlation was used to examine associations between structural indicators and semantic scores, with the aim of identifying where the two sources of evidence converged or diverged across SSDA dimensions. A principal component analysis (PCA) was applied to the ten network indicators to produce a summary index for comparison with semantic scores. The purpose of applying PCA here was not to identify latent factors but to generate a single structural complexity score for plotting against semantic scores. This composite score served a different purpose from the dimension-specific correlations, providing a single axis for quadrant-based classification of learner profiles across the full structural-semantic space.

This score and the total semantic score were then plotted against each other, and the four quadrants formed by the two mean lines served to distinguish structural–semantic profile types. Representative cases were then selected from each profile. These were analyzed in detail using the original concept maps and written records to illustrate how the profiles differed in their structural and semantic characteristics.

The structural indicators and semantic scores in SSDA are theoretically mapped onto its three dimensions, but this mapping does not by itself establish construct validity, and whether the two indeed capture different facets of systems thinking is one of the questions this study sets out to examine through structural–semantic comparison. The correlations, mismatches, and profile types reported in the Results section should therefore be read as descriptive evidence about what each type of indicator captures. SSDA is offered here as an exploratory framework, and its broader applicability awaits further investigation.

### 3.8 Supplementary causal-chain completeness analysis

To further examine the relationship between structural complexity, link validity, and semantic quality, we conducted a supplementary causal-chain completeness analysis alongside the SSDA analysis. This analysis differed from the primary expert semantic ratings by focusing on whether the directional links represented in the concept maps were valid and whether they formed continuous mechanism chains. This design was informed by causal-map research that evaluates map quality through the correctness and depth of chained causal links (Jeong, 2020; Shin and Jeong, 2021).

Two raters constructed six expert reference chains based on the Lop Nur task material, corresponding to historical hydrological change and oasis decline, modern upstream development and downstream ecological degradation, potash development and resource production, the tension between potash extraction and ecological restoration, ecological degradation feedback, and cross-spatiotemporal water governance. Each reference chain was scored on a 0–3 scale, with 0.5-point intervals allowed. Distal causal shortcuts such as A → C or A → D received partial credit when the direction was broadly plausible but a key intermediate mechanism was omitted. The full scoring rules and expert reference chains are provided in Appendix C.

Two raters independently scored all 47 concept maps for causal-chain completeness. Inter-rater reliability was examined using quadratic weighted Cohen’s kappa and intraclass correlation coefficients. The weighted kappa values for the six reference chains ranged from 0.697 to 0.862, and the overall weighted kappa across all chain-level ratings was 0.814. The ICC(2,1) for the total chain-completeness score was 0.837, indicating acceptable inter-rater reliability. For the formal analysis, the final score for each reference chain was calculated as the mean of the two raters’ scores. The six averaged chain scores were summed to obtain the Chain Completeness Score and then converted to a normalized 0–100 score.

## 4 Results

### 4.1 Overall performance: adequate information coverage but weak representation of feedback mechanisms

Table 3 summarizes the descriptive statistics of the concept maps. On average, the concept maps included a substantial number of elements, with a mean node count of 17.79, above the suggested minimum of 15 concept nodes in the task instructions. Structurally, however, these networks were generally sparse (density = 0.067).

**Table 3:** Descriptive statistics of network structural indicators and semantic scores for preservice teachers’ concept maps (*N* = 47).

Dimension	Variable	Mean	SD	Median	Min	Max
Structural metrics	Node count	17.79	5.09	17	8	36
	Edge count	18.62	6.45	18	6	38
	Density	0.067	0.026	0.062	0.03	0.167
	Avg. degree	1.04	0.2	1.04	0.6	1.83
	Diameter	7.28	2.9	7	3	15
	Num. of cycles	1.34	1.36	1	0	5
	Reciprocity	0.089	0.165	0	0	1
	ST label count	16.4	6.23	17	0	35
	ST label ratio	0.91	0.2	1	0	1
	Cross-ST edges	6.06	3.85	5	0	16
Semantic scores	Dimension A	2.47	0.6	2.5	1	3
	Dimension B	2.4	0.62	2.5	1	3
	Dimension C	2.66	0.43	3	2	3
	Total score	7.53	1.31	7.5	4	9

**Figure 3:**
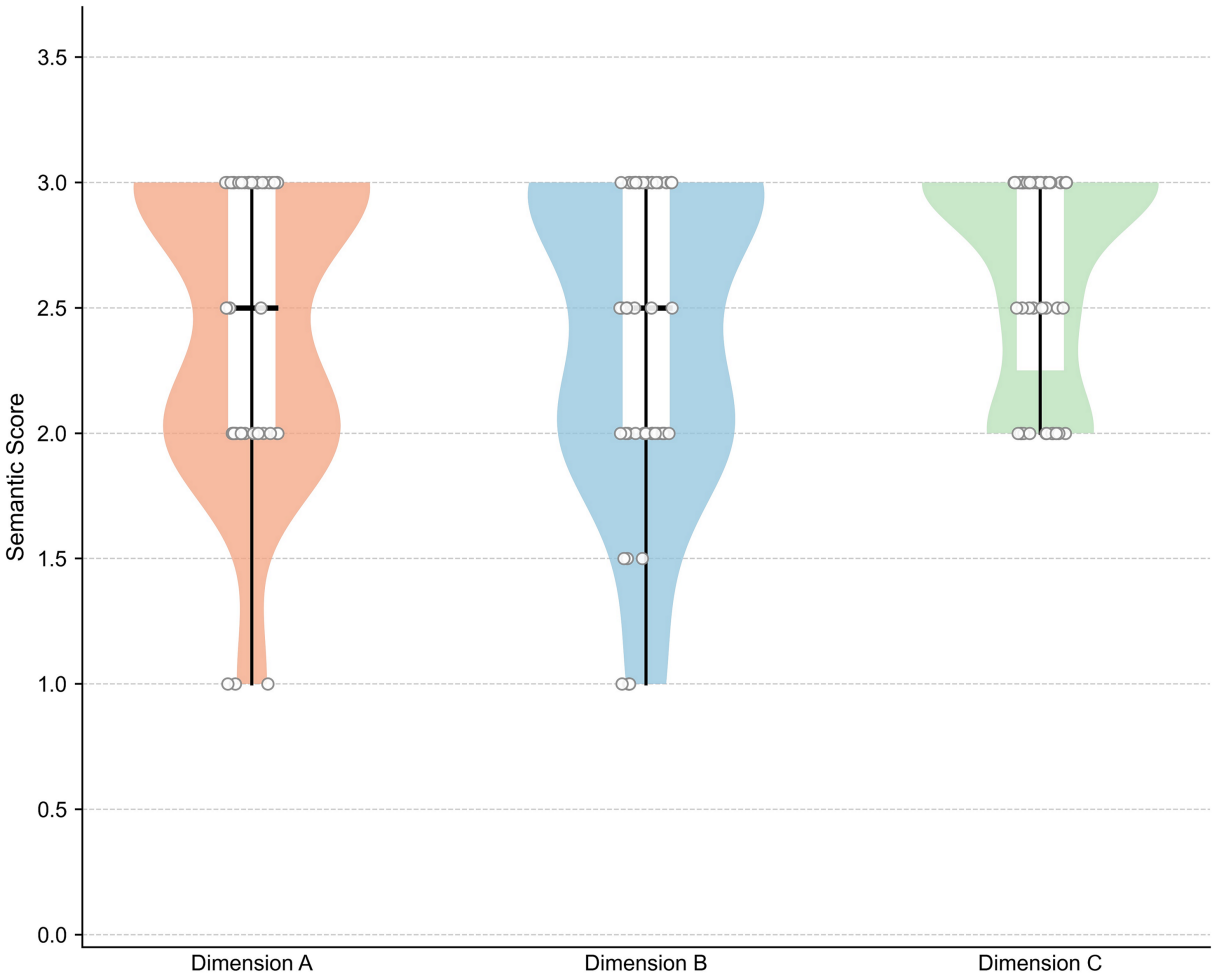
Distribution of semantic scores across three dimensions. Violin plots display the score distribution for each SSDA dimension (*N* = 47): Dimension A—Network Structuring (pink), Dimension B—Nonlinear Mechanism Deconstruction (blue), and Dimension C—Spatiotemporal Reasoning (green). The embedded box plots show the median (thick bar) and interquartile range; open circles represent individual participant scores.

Semantic scores varied across the three dimensions (Figure 3). The average score for Dimension C (Spatiotemporal Reasoning) reached 2.66, with scores primarily concentrated in the upper range. Dimension A (Network Structuring) exhibited a roughly normal distribution of scores (M = 2.47), with fewer participants achieving a perfect score compared to Dimension C. Dimension B (Nonlinear Mechanism Deconstruction) showed the lowest average score (M = 2.40), with the largest gap between high and low performers.

Comparing structural and semantic evidence showed the most pronounced mismatch in Dimension B (Nonlinear Mechanism Deconstruction). Judging by the written records, some participants were able to describe feedback mechanisms, identify cyclic relationships, and recognize interactions among elements. However, in terms of structural indicators, the average cycle count was only 1.34, and the average reciprocity score was 0.089.

### 4.2 Correlations between structural indicators and semantic scores

**Figure 4:**
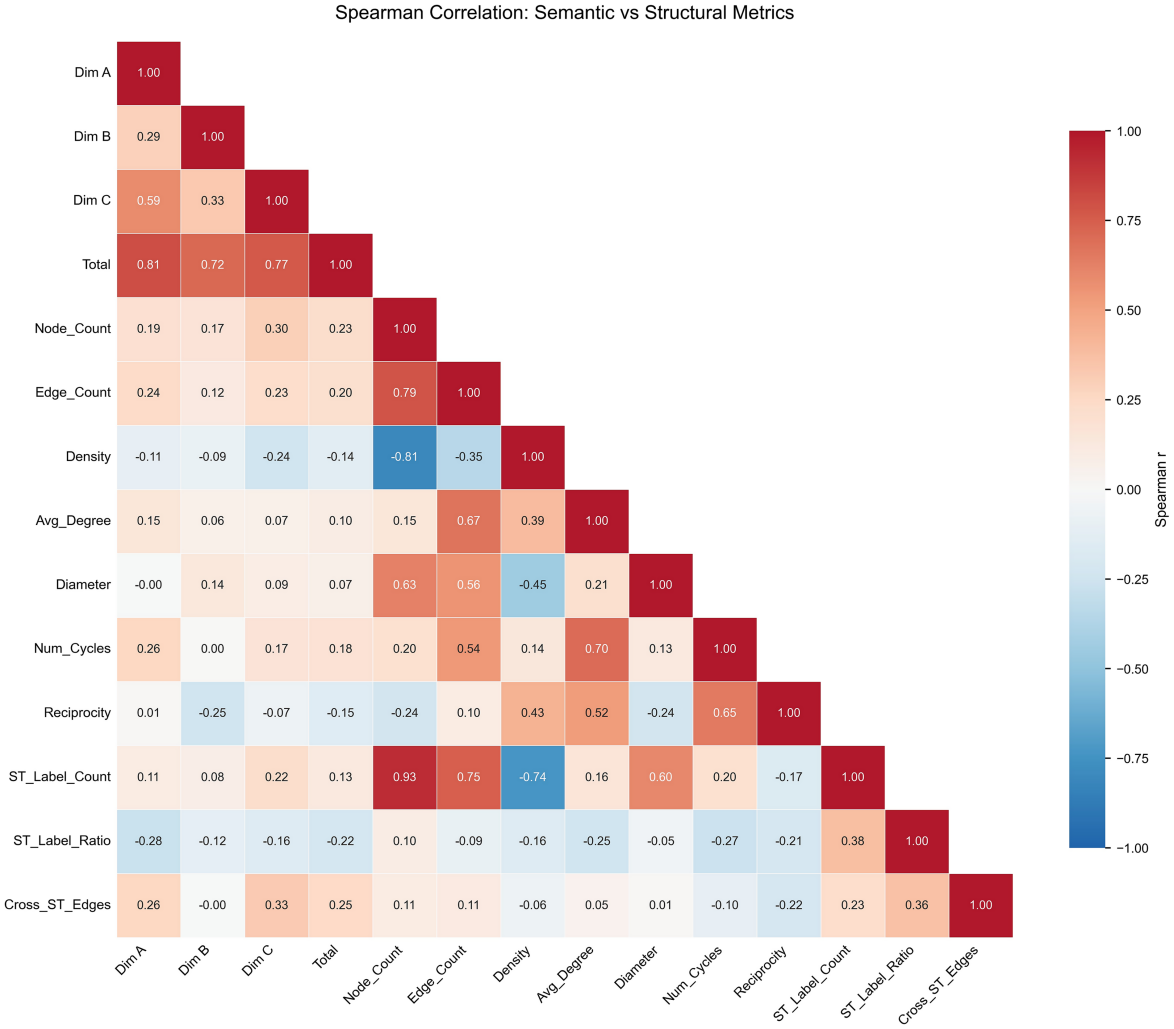
Correlations between structural indicators and semantic scores. The heatmap displays Spearman correlation coefficients between all pairs of structural indicators and semantic scores (*N* = 47). Red cells indicate positive correlations and blue cells indicate negative correlations; color intensity reflects the magnitude of the coefficient. Numerical values are shown within each cell. Dim A/B/C: semantic scores for Dimensions A, B, and C of the SSDA framework; Total: total semantic score; ST: spatiotemporal.

Several structural indicators (e.g., cycle count, reciprocity) were concentrated at low values with non-normal distributions, and the semantic scores were ordinal. Spearman’s rank correlation was therefore used to examine relationships among variables (de Winter et al., 2016). The full correlation matrix is shown in Figure 4.

Node count, edge count, and spatiotemporal label count were highly intercorrelated (*ρ* = 0.75–0.93), yet all three showed weak correlations with semantic scores across dimensions (ρ = 0.08–0.30). Density showed strong negative associations with both node count (ρ = −0.81) and spatiotemporal label count (ρ = −0.74), indicating that larger maps tended to have lower connection density.

Dimension B showed the sharpest disconnect between structural indicators and semantic scores. Cycle count was virtually uncorrelated with mechanistic semantic scores (ρ = −0.001); reciprocity showed minimal variation across the sample.

Structural indicators for Dimension C showed stronger associations with semantic scores. The number of cross-spatiotemporal links showed a moderate correlation with Dimension C semantic scores (ρ = 0.327), the strongest structural-semantic correspondence observed across the three dimensions. The raw count of spatiotemporal labels, by contrast, was much more weakly associated with semantic scores.

### 4.3 Systems thinking types based on structural-semantic profiles

Principal component analysis of the ten network indicators yielded one component with an eigenvalue above 1, accounting for 39.05% of the total variance (KMO = 0.593, Bartlett’s test *p* < 0.001). Component loadings are reported in Table 4.

**Table 4:** Component loadings of structural indicators on the first principal component.

Structural indicator	Component loading
Node count	0.948
Edge count	0.872
Density	−0.649
Avg. degree	0.127
Diameter	0.734
Num. of cycles	0.337
Reciprocity	−0.275
ST label count	0.948
ST label ratio	0.393
Cross-ST edges	0.169

The highest loadings were observed for node count (0.948), spatiotemporal label count (0.948), edge count (0.872), and graph diameter (0.734). This component thus primarily captured the scale and structural span of the concept maps. Density loaded negatively (−0.649), indicating that larger concept maps tended to have lower connection density, consistent with the sparse network pattern observed in the correlation analysis above.

**Figure 5:**
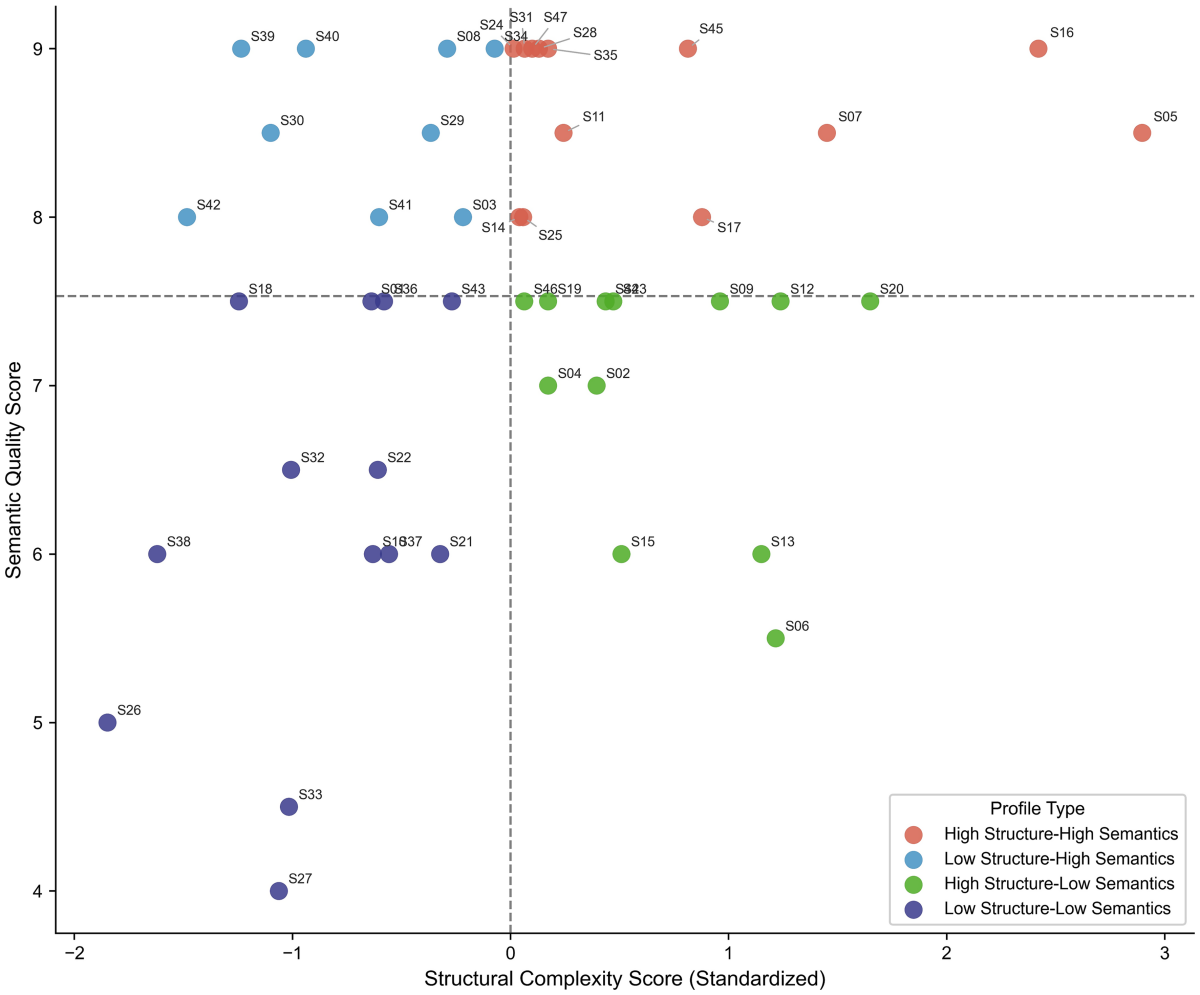
Scatter plot of systems thinking profiles based on structural complexity and semantic scores. Each point represents one participant (*N* = 47), labeled with participant code. The x-axis shows the standardized first principal component score derived from ten structural indicators; the y-axis shows the total semantic score. Dashed lines indicate sample means on each axis, dividing participants into four structural–semantic profile types: High Structure–High Semantics (HS-HS, red), Low Structure–High Semantics (LS-HS, cyan), High Structure–Low Semantics (HS-LS, green), and Low Structure–Low Semantics (LS-LS, dark blue).

A scatter plot was constructed with the principal component score on the horizontal axis and the total semantic score on the vertical axis (Figure 5). Using the sample means on both dimensions as dividing lines, participants were distributed across four quadrants, forming four structural-semantic profile types (Table 5). The four profile types were labeled as HS-HS (High Structure–High Semantics), LS-HS (Low Structure–High Semantics), HS-LS (High Structure–Low Semantics), and LS-LS (Low Structure–Low Semantics).

**Table 5:** Structural-semantic profile types and their mean scores.

**Type**	***n***	**Structural score (M)**	**Semantic score (M)**
HS-HS (High Structure–High Semantics)	13	0.71	8.65
LS-HS (Low Structure–High Semantics)	9	−0.70	8.56
HS-LS (High Structure–Low Semantics)	12	0.70	7.00
LS-LS (Low Structure–Low Semantics)	13	−0.88	6.19

Sample sizes ranged from 9 to 13 across the four types, indicating a reasonably balanced distribution. Two comparisons stand out in Table 5. HS-HS and HS-LS had nearly identical structural scores (0.71 vs. 0.70) but markedly different semantic scores (8.65 vs. 7.00), indicating that concept maps of similar structural complexity can differ substantially in semantic scores. HS-HS and LS-HS had very similar semantic scores (8.65 vs. 8.56) but vastly different structural scores (0.71 vs. –0.70), suggesting that these two groups obtained comparable semantic scores via markedly different structural configurations. LS-LS performed poorly on both measures (structural score: −0.88; semantic score: 6.19). Given the modest sample size, these four profiles are best understood as descriptive patterns rather than definitive categories, and their boundaries should be interpreted with appropriate caution.

### 4.4 Causal-chain completeness as supplementary evidence

The supplementary causal-chain completeness analysis further examined the relationship among structural complexity, link validity, and semantic quality. The normalized chain-completeness score was significantly correlated with the original total semantic score (Spearman’s *ρ* = 0.563, *p* < 0.001). It was also significantly correlated with the structural principal component score (ρ = 0.550, p < 0.001). This indicates that the chain-completeness score depended on the structural support provided by the concept maps, while also reflecting whether the represented links were valid and whether the mechanism chains were continuous. It therefore served as supplementary evidence linking structural indicators and semantic ratings.

The relationships between each reference chain and the original total semantic score were not the same (Table 6). C1, historical hydrological change and oasis decline; C2, modern upstream development and downstream ecological degradation; and C3, potash development and resource production, showed weak correlations with the total semantic score, suggesting that these chains mainly reflected participants’ coverage of explicit information in the task material. In contrast, C4, the tension between potash extraction and ecological restoration (ρ = 0.483, *p* < 0.001); C5, ecological degradation feedback (ρ = 0.437, *p* < 0.01); and C6, cross-spatiotemporal water governance (ρ = 0.543, *p* < 0.001), showed stronger correlations with the total semantic score. This suggests that semantic quality was not distinguished by the number of concepts or general causal relations alone; stronger differentiation appeared when learners organized development–restoration tensions, feedback mechanisms, and cross-spatiotemporal governance relations into valid mechanism chains.

**Table 6:** Correlations between supplementary causal-chain completeness scores and the original total semantic score.

Measure	Spearman’s ρ	*p* value
Normalized chain-completeness score	0.563	<0.001
C1 Historical hydrological change and oasis decline	0.062	0.677
C2 Modern upstream development and downstream ecological degradation	0.142	0.341
C3 Potash development and resource production	0.091	0.541
C4 Potash extraction–ecological restoration tension	0.483	<0.001
C5 Ecological degradation feedback	0.437	<0.01
C6 Cross-spatiotemporal water governance	0.543	<0.001

The four structural–semantic profile types also differed in their chain-completeness scores. The HS-HS group had the highest mean normalized chain-completeness score (M = 72.9), whereas the LS-LS group had the lowest score (M = 53.0), and the group difference was significant (Kruskal–Wallis *p* < 0.001). The LS-HS group (M = 61.0) and the HS-LS group (M = 62.8) had similar total scores, suggesting that a larger structural scale could lead to broader chain coverage but did not necessarily indicate higher-quality key mechanism chains. At the chain level, the LS-HS group scored slightly higher than the HS-LS group on C5 and C6, indicating that some structurally concise maps still represented key feedback and cross-spatiotemporal governance chains. Together, these results show that chain completeness helps explain why structurally rich maps and semantically strong maps did not always coincide.

### 4.5 Structural-semantic contrasts in representative cases

This study selected three representative cases for further comparative analysis. Among them, S05 (HS-HS, High Structure–High Semantics) served as the baseline case because this participant scored high on both structural and semantic measures. The other two cases were S02 (HS-LS, high structure–low semantic) and S03 (LS-HS, low structure–high semantic). By contrasting these two different types of structure–semantic mismatch, the study analyzes the underlying reasons for the discrepancies. For readability and participant privacy, the concept maps presented in this paper are transcribed versions in which node labels and link descriptions have been translated from Chinese into English, while all other structural information has been preserved from the originals.

#### 4.5.1 S05 (HS-HS): structure and mechanism in alignment

**Figure 6:**
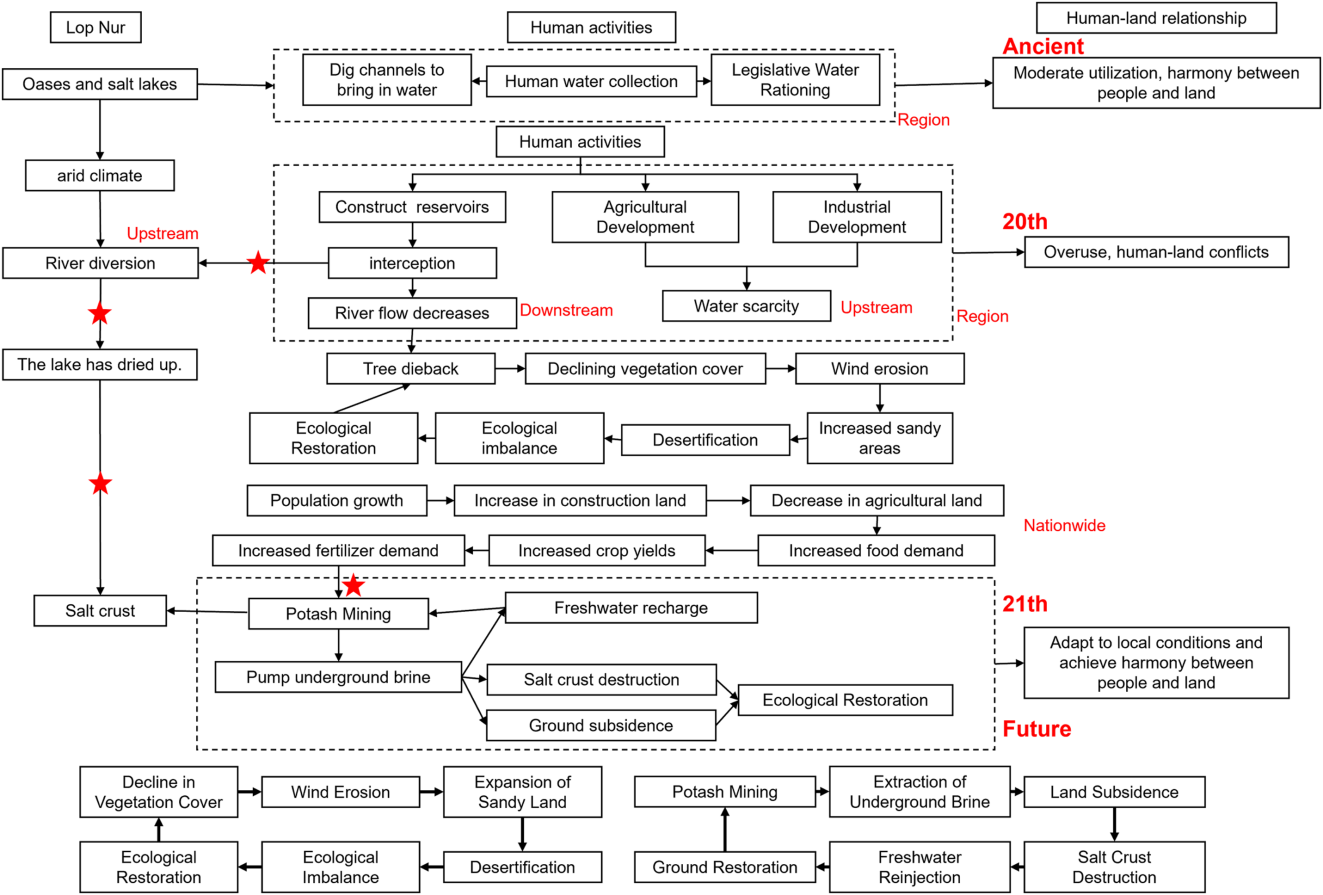
Transcribed concept map of participant S05 (High Structure–High Semantics type). Scores: structural metrics: nodes = 36, edges = 38, density = 0.0302, cycles = 2, cross-spatiotemporal links = 4, structural PCA score = 2.90; semantic rubric scores: Dimension A = 3, Dimension B = 2.5, Dimension C = 3, total = 8.5; normalized chain-completeness score = 81.9. The map is organized into thematic modules separated by dashed boxes according to temporal period (Ancient, 20th century, 21st century, Future). Red text indicates spatial scale annotations (Upstream, Downstream, Region, Nationwide); red stars mark cross-spatiotemporal connections identified by the participant. The right panel contains the participant’s written characterizations of human–land relationships across temporal periods, and the bottom panel lists the participant’s written descriptions of identified feedback mechanisms, both corresponding to the supplementary written task. Node labels and link descriptions have been translated from Chinese into English; all structural features are preserved from the original.

S05’s concept map contained a relatively high number of nodes and links within the sample, while retaining a clear visual organization (Figure 6). The participant grouped the information into several distinct sections, including “Human Activities and Water Resource Changes,” “Vegetation Degradation and Land Desertification,” and “Salt Crust Extraction and Surface Deformation.” Each section was organized around a core process, and causal chains connected the sections to one another. Despite the large amount of information, the hierarchical structure remained clearly discernible.

Within each section, the participant also labeled causal relationships with some specificity. One loop clearly depicted a positive feedback process in which vegetation loss leads to stronger wind erosion, accelerating desertification and further reducing vegetation, and this loop was drawn as a closed cycle in the map. The second pathway started with salt mining and proceeded through brine extraction, land subsidence, salt crust damage, and freshwater reinjection, linking resource extraction with restoration-related intervention. This pathway was described verbally and was also partially reflected in the concept map, though it did not successfully close into a complete loop (Dimension B = 2.5). The map’s spatiotemporal labels were comprehensive, earning it a score of 3 in Dimension C.

In the final map, the content appeared as several clearly delineated modules, with space between modules used for causal annotations. Mechanistic details were represented within each module. This organization gave the concept-map structure explanatory value and helped make the participant’s reasoning visible. The supplementary chain-completeness score supported this interpretation: S05 received a normalized score of 81.9, the highest among the three cases, with full scores on C2 and C5 and a near-full score on C4.

#### 4.5.2 S02 (HS-LS): much information but little reorganization

**Figure 7:**
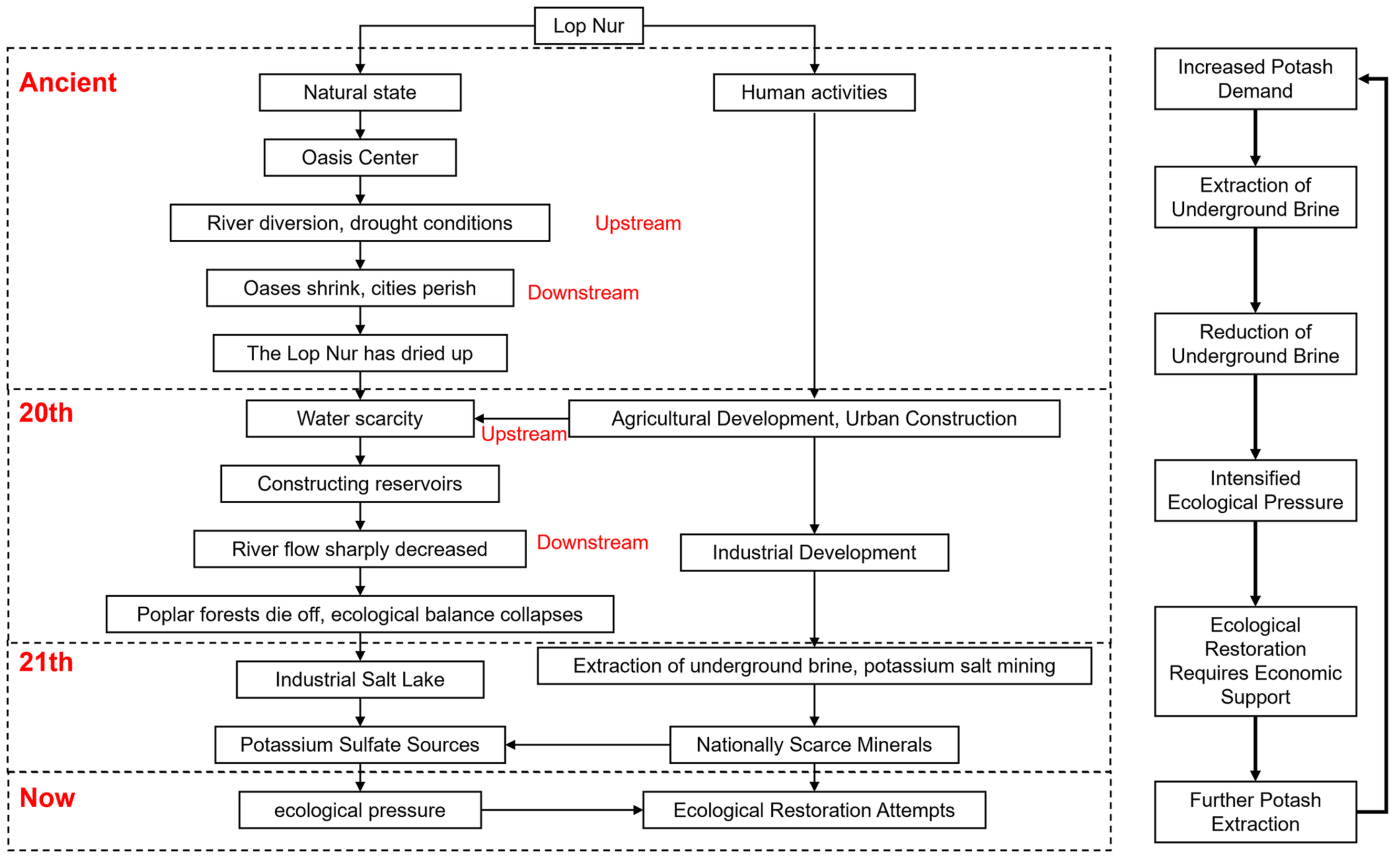
Transcribed concept map of participant S02 (High Structure–Low Semantics type). Scores: structural metrics: nodes = 19, edges = 20, density = 0.0585, cycles = 0, cross-spatiotemporal links = 8, structural PCA score = 0.39; semantic rubric scores: Dimension A = 2, Dimension B = 3, Dimension C = 2, total = 7.0; normalized chain-completeness score = 52.8. The map extends from the central “Lop Nur” node into two parallel vertical lines representing natural processes (left) and human activities (right), organized by temporal period (Ancient, 20th century, 21st century, Now) separated by dashed lines. Red text indicates spatial scale annotations (Upstream, Downstream). Lateral connections between the two lines are sparse and lack relationship labels. The right panel contains the participant’s written descriptions of identified feedback mechanisms, corresponding to the supplementary written task. Node labels and link descriptions have been translated from Chinese into English; all structural features are preserved from the original.

S02’s concept map was rich in nodes and featured complete spatiotemporal labels. With a structural score of 0.39, it fell into the high-structure category (Figure 7). This case was classified as HS-LS based on the overall structural and total semantic scores; within this overall profile, Dimension B shows a more specific mismatch between the participant’s written explanation and the graphical representation.

However, this structural abundance did not translate into stronger relational organization. The concept map started from the “Lop Nur” node and extended into two parallel main lines, pointing to natural conditions and human activities, respectively. There were very few lateral connections between the two lines, and they lacked relationship labels. The overall layout followed the timeline provided by the material, showing limited classification or reorganization of concepts (Dimension A = 2). Although spatiotemporal labels were present, they mainly served to categorize nodes rather than to establish clear cross-spatiotemporal causal links (Dimension C = 2).

In Dimension B (feedback mechanisms), the participant demonstrated the most pronounced semantic–structural mismatch. In the written narrative, the participant presented a complete feedback loop with clear stepwise causal logic, illustrating the cyclical relationship between economic development and ecological degradation. However, in the concept map, the participant failed to effectively present these relationships. Nodes such as “potash mining” were located in the lower part of the human activities line, while “ecological pressure” and “ecological restoration” were near the bottom, with no effective connections formed between these nodes. The supplementary chain-completeness score showed the same limitation. S02 received a normalized score of 52.8, lower than S03; its low C5 score and modest C3, C4, and C6 scores indicate that many of its links remained local or mechanistically compressed.

#### 4.5.3 S03 (LS-HS): a simple map with strong reasoning

**Figure 8:**
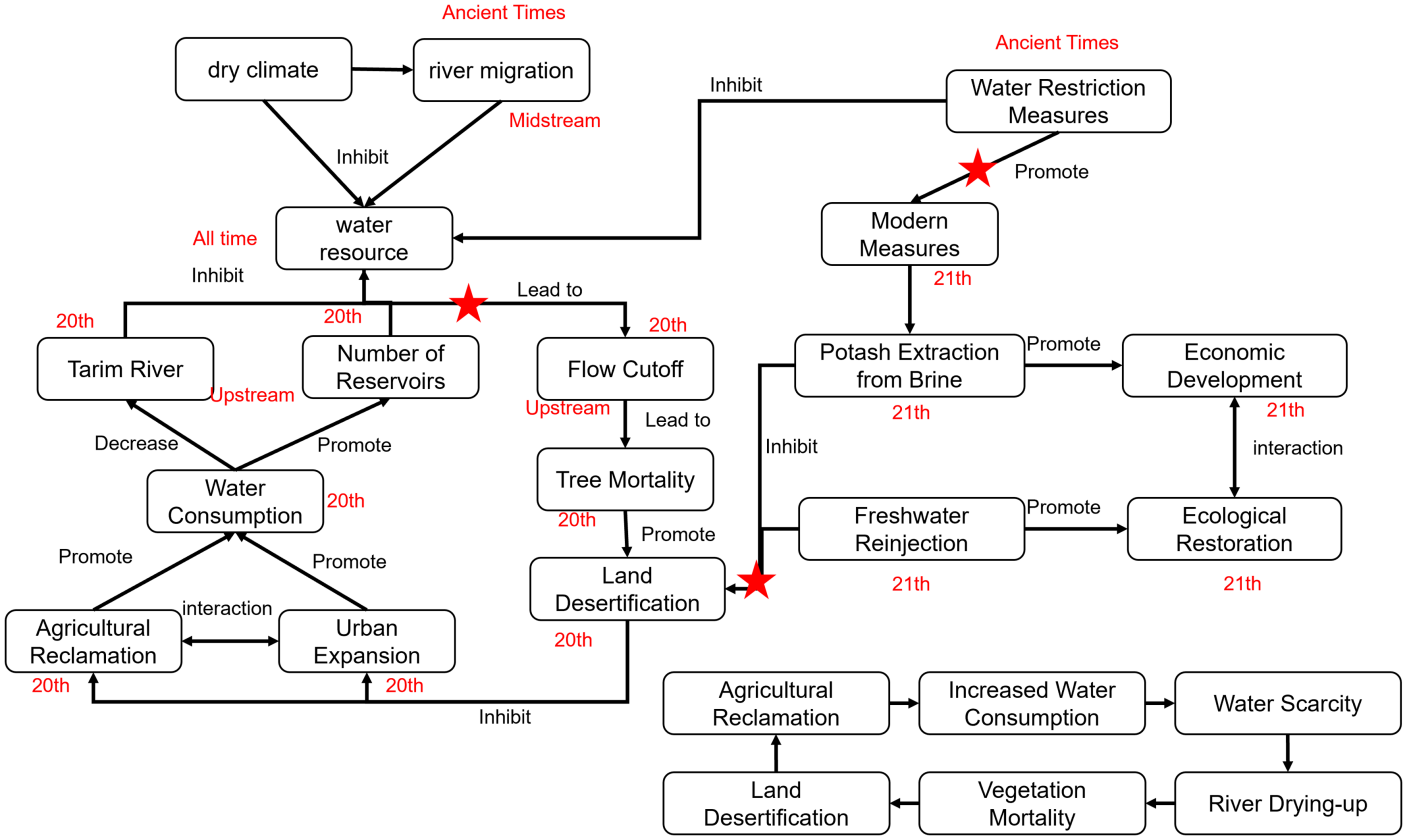
Transcribed concept map of participant S03 (Low Structure–High Semantics type). Scores: structural metrics: nodes = 16, edges = 23, density = 0.0958, cycles = 2, cross-spatiotemporal links = 5, structural PCA score = −0.22; semantic rubric scores: Dimension A = 3, Dimension B = 3, Dimension C = 2, total = 8.0; normalized chain-completeness score = 58.3. Rather than a linear timeline, the map distributes nodes across the space with temporal annotations attached to individual nodes (Ancient Times, 20th century, 21st century, All time) and spatial scale annotations (Upstream, Midstream) in red text. Red stars mark cross-spatiotemporal connections identified by the participant. The bottom-right panel lists the participant’s written descriptions of identified feedback mechanisms, corresponding to the supplementary written task. Node labels and link descriptions have been translated from Chinese into English; all structural features are preserved from the original.

S03’s concept map was constructed around only a few core causal chains (Figure 8), resulting in a relatively low structural score (−0.22).

The map followed a single main thread, moving from human activities to natural processes and then on to system outcomes. Most links were labeled with specific relationship terms such as “leads to,” “intensifies,” and “inhibits,” so causal directions and the nature of each interaction were easy to read. Despite the small number of nodes, the concepts were organized with a coherent internal logic, earning a Dimension A score of 3.

The map contained several visible feedback structures. The lower half of the map contained a complete closed loop. Agricultural reclamation and urban expansion led to increased water consumption, then water scarcity, river drying-up, tree mortality, and land desertification, with an “inhibits” arrow looping back from desertification to agricultural reclamation and urban expansion. A second “inhibits” arrow ran from land desertification all the way up to the water resources node at the top of the map, creating a long-range feedback link that cut across several levels. The map also marked a bidirectional “mutual interaction” between agricultural reclamation and urban expansion. In the written record, the participant described this feedback loop again, consistent with the structure shown in the map (Dimension B = 3). In terms of spatiotemporal information, key processes were arranged chronologically, from early environmental changes through subsequent human activities to recent restoration efforts (Dimension C = 2). This concentration on a few core chains was also reflected in the supplementary score. S03 received a normalized chain-completeness score of 58.3, higher than S02, with full scores on C2 and C5.

The case of S03 shows that high-quality semantic expression can coexist with a concise structure. Compared with S02, S03 represented mechanistic reasoning more effectively through relation labels and feedback structures in the concept map.

## 5 Discussion

Examining structural indicators and semantic scores together made the structural–semantic mismatches in participants’ concept maps more visible. For instance, HS-HS (High Structure–High Semantics) and HS-LS (High Structure–Low Semantics) had similar structural scores (0.71 vs. 0.70) but clearly different semantic scores (8.65 vs. 7.00). This comparison suggests that structural indicators alone are insufficient for interpreting systems thinking as represented in concept maps. Addressing the three research questions raised in the introduction, the following sections examine: what patterns characterize preservice STEM teachers’ structural and semantic performance (RQ1); under what circumstances structural indicators correspond to semantic scores and where they fall short (RQ2); and what the diversity of structural–semantic profiles implies for STEM teacher preparation (RQ3).

### 5.1 What structural indicators can and cannot capture

It has long been observed that the structural complexity of a concept map does not necessarily indicate the semantic quality of the represented understanding (Gray et al., 2019; Khajeloo and Siegel, 2022). This study reaffirms that view. However, by comparing differences across dimensions, the research further reveals that the degree of this structure–semantic mismatch varies among dimensions. Dimension C (Spatiotemporal Reasoning) showed the highest degree of match (*ρ* = 0.327), followed by Dimension A. In contrast, for Dimension B, the match between the two approached zero.

What accounts for this difference? It may be because the cognitive processes each dimension engages differ in how visible they are on a concept map. Spatiotemporal labels can be directly observed and counted on the map, making them more easily captured by structural indicators. However, feedback mechanisms rely more on internal causal reasoning; even if a learner understands the cyclic relationships between variables, they may not necessarily be able to present them in the diagram. The hierarchical layout that most concept maps default to is suited for taxonomic knowledge, not the dynamic causal relationships represented by feedback loops (Safayeni et al., 2005). Therefore, the more pronounced structure–semantic mismatch in Dimension B is also related to the inherent limitations of concept maps themselves.

It should be clarified that this study does not treat semantic ratings as a privileged ground truth. Rather, semantic ratings represent expert judgments of link validity and explanatory coherence, whereas structural indicators represent the formal organization of the concept map. Either source of evidence may appear stronger or weaker depending on the representational demands of the task.

Another possibility is that the ability to transform and shift between different external representations is itself a distinct cognitive demand (Pande and Chandrasekharan, 2017). The concept map created by participant S02 serves as a typical example. The participant’s written account described a complete feedback loop. On the concept map, however, the nodes that should have formed this loop were scattered, with no connecting lines closing them into a circuit. For S02, the structural–semantic mismatch in Dimension B likely stems from difficulties encountered in the process of translating verbal description into diagrammatic representation. This is because, when presenting cyclic relationships through oral description or written response, one only needs to consider the connection from the current step to the next—a localized, linear task. In contrast, representing it in a diagram requires the learner to simultaneously consider the spatial relationships of elements, the direction of arrows, and whether the loop actually closes (Cook, 2006).

The typological results make this point more concrete. HS-HS and HS-LS showed almost identical structural scores but differed clearly in semantic scores; an assessor relying only on structural scores would therefore treat the two groups as comparable and would also risk underestimating LS-HS maps, which were structurally concise but semantically strong. The supplementary chain-completeness analysis further clarifies this mismatch. The normalized chain-completeness score was significantly correlated with both the total semantic score and the structural principal component score, suggesting that valid mechanism chains require structural support while also depending on link validity and chain continuity. The stronger associations between C4, C5, C6 and the total semantic score further indicate that development–restoration tension, feedback relations, and cross-spatiotemporal governance captured understanding quality more clearly than general content coverage.

The PCA results add a methodological constraint to this interpretation: node count and edge count dominated the structural principal component, whereas cycle count and reciprocity contributed less. The structural principal component explained only 39.05% of the variance, indicating that concept-map structure itself was multidimensional. Thus, a single structural index may describe the external scale and organizational span of a concept map, but it should not be used to directly infer the quality of learners’ systems understanding (Ruiz-Primo and Shavelson, 1996). This point may also apply to other STEM contexts in which concept maps are used to assess systems thinking.

The differences in structural–semantic correspondence reported above should be interpreted as task-specific evidence. They do not provide a general judgment on the performance of structural indicators in concept-map assessment. The stability of SSDA across other tasks, disciplines, and learner populations requires further investigation.

### 5.2 Task context and disciplinary cues shape spatiotemporal reasoning in STEM sustainability assessment

Dimension C scored highest semantically (M = 2.66) and showed the strongest structural–semantic correlation (*ρ* = 0.327). The Lop Nur task materials contained clear temporal trajectories and spatial scale contrasts, offering learners ready-made cues for identifying cross-scale causal relationships. As a discipline situated within the broader STEM spectrum of Earth and environmental sciences, geography may have made participants more attentive to such cues. Research in geography education has also found that enriching causal diagrams with scale information can effectively promote students’ systems thinking performance (Cox et al., 2020). Conversely, when causal events are separated in time or space and the task offers no explicit cues, learners struggle to connect them across scales (Khine and Saleh, 2013). The relatively high performance on Dimension C therefore appears to be more closely related to the alignment between the task’s spatiotemporal cues and the cross-scale reasoning this dimension assesses than to participants’ disciplinary background alone.

This pattern may have relevance beyond the Lop Nur case, given that causal processes in sustainability issues often span multiple temporal and spatial scales (Martín-López et al., 2020), placing particular demands on how learners organize and represent information (Nyerges et al., 2014). Many misconceptions about sustainability arise precisely because learners cannot shift between the relevant temporal and spatial scales (Skarstein and Wolff, 2020). When assessment tasks make the spatiotemporal structure of the topic visible, cross-scale reasoning gains a foothold; without such cues, the same reasoning may remain hidden for want of a cognitive anchor (Hovardas et al., 2022). This aligns with GreenComp’s identification of “understanding interactions across time, space, and context” as central to systems thinking (European Commission and Joint Research Centre, 2022). For STEM sustainability education more broadly, this suggests that assessment tasks which make the spatiotemporal structure of a problem explicit may better elicit cross-scale reasoning across disciplinary contexts.

### 5.3 Representational patterns associated with higher-level systems thinking

The case analyses showed that structurally similar concept maps could differ clearly in semantic quality. This difference appeared in how information was organized in the final concept maps. Some maps contained many nodes and relatively complete spatiotemporal annotations, yet the overall organization still largely followed the sequence of the source material. Relations among concepts were weakly specified, and the map as a whole read more as a reproduction of the material than as a reorganization of it. Other maps organized information into functional modules or a small number of core causal chains. These maps showed clearer causal connections between modules, and feedback structures could also be represented as closed loops. The chain-completeness analysis also showed that the chains more strongly associated with semantic quality were the key mechanism chains represented by C4, C5, and C6.

These representational patterns are consistent with prior research on concept-map quality and complex systems understanding. Concept maps associated with deep learning tend to show more integrated structures and clearer relational explanations, whereas concept maps associated with surface learning often remain at the level of information listing (Hay, 2007). Research on complex systems understanding similarly shows that learners with weaker understanding tend to represent systems in static and fragmented ways, whereas stronger learners identify interactions among system components and distinguish between levels of analysis (Raia, 2005). The SBF framework provides another way to interpret this difference: in SBF terms, lower-level representations remain mainly at the structural level, listing what elements a system contains, whereas higher-level representations move toward the behavioral level by focusing on how these elements interact (Hmelo-Silver, 2004).

This study analyzed representational outcomes in the final concept maps rather than real-time cognitive strategies during map construction. We therefore treated “functional modular organization” and “core-chain organization” as representational patterns visible in the final maps. This distinction matters for assessment because criteria that privilege one mapping style may underestimate learners who represent key mechanism chains through more concise structures. The SSDA framework and the chain-completeness score therefore help assessors distinguish the scale of a map from the depth of conceptual understanding.

### 5.4 Implications for STEM teacher preparation in sustainability education

STEM teacher education programs often emphasize instructional design or content knowledge, with less explicit attention to preservice teachers’ assessment competencies, particularly in evaluating students’ systems thinking in complex sustainability contexts (Fischer et al., 2022). The structural-semantic disconnect, the influence of task context on performance, and the diversity of structural–semantic profiles observed in this study are all issues that assessors need to recognize and navigate in practice. The SSDA framework developed in this study is not intended as a rapid classroom grading tool; rather, it is better suited as a research instrument and diagnostic training tool through which preservice teachers learn to assess systems thinking in concept maps. When preservice teachers use this framework to assess concept maps, they need to distinguish structural features from semantic quality across dimensions and judge whether the two are aligned. This process may support the development of professional judgment in systems thinking assessment. Sustainability issues are closely connected to the content and inquiry tasks that preservice teachers will later teach. Embedding SSDA-based training in discussions of such issues allows assessment literacy and disciplinary understanding to develop in tandem. Such integration holds particular promise for STEM teacher preparation programs that aim to build both sustainability competence and evaluative judgment simultaneously.

### 5.5 Limitations and future research

The findings reported here are based on 47 preservice teachers in a STEM-related discipline working with a single case, the evolution of the Lop Nur human–land system. This design ensured content consistency, but also limited the transferability of the results. As discussed earlier in the Disciplinary Context section, the strong spatiotemporal structure of the Lop Nur case likely contributed to the high performance on Dimension C. Whether the same patterns would hold for sustainability topics with less pronounced spatiotemporal structure, such as food safety, social equity, energy policy trade-offs, or biology-related topics such as ecosystem dynamics and food webs, remains an open question.

Concept maps are themselves highly sensitive to task design parameters. Different concept-mapping approaches—including the medium of construction and the level of guidance—have been shown to substantially affect the level of systems thinking reflected in the resulting maps (Brandstädter et al., 2012). The building strategies learners adopt during construction can also shape the structural quality of the final map (Srivastava et al., 2021). The present study used a 45-min time limit, predefined source material, and structured scaffolding prompts. This configuration may not readily prompt learners to work feedback relationships into closed-loop structures. The absence of dedicated training in system-oriented concept mapping may have affected participants’ ability to translate feedback mechanisms into closed-loop graphical structures. Future quasi-experimental studies could examine this possibility by comparing learners who receive explicit training in representing feedback structures with those who complete the task under the scaffolded, non-training condition used here.

Two features observed in the Results align with this characterization: a relatively high node count combined with low network density, and feedback mechanisms that appeared only in the written records without forming closed loops in the maps. Under a different task design—for example, with a dynamic focus question, a longer time allowance, or scaffolds specifically targeting feedback structures—learners’ Dimension B structural scores, as well as the overall relationship between structural and semantic scores, could differ. Whether the patterns reported here would be observed under other task conditions therefore awaits further investigation.

Instructional conditions also shape the maps participants produce. An intervention study has shown that explicitly introducing spatial scale into causal diagrams substantially changes how students represent systems thinking, with the experimental group attending more systematically to cross-scale interactions than the control group (Cox et al., 2020). The participants in the present study had not received any dedicated training in systems thinking before the data collection. The structural–semantic patterns observed here should therefore be interpreted in relation to participants’ prior learning experiences and the absence of such targeted instruction. Had participants received prior training, particularly training that targeted the recognition and construction of feedback structures, their Dimension B performance might have departed from the patterns reported here. This suggests that participants’ prior learning experiences should be taken into account when interpreting the structural–semantic relationships observed in any given sample.

Sample size is a further constraint, particularly for the typological classification. With 47 participants split into four quadrants and the smallest group numbering only 9, the statistical stability of these categories is limited. A larger sample might yield a more nuanced typology and, crucially, groups large enough for statistical comparison.

The methodological tools used in this study also have limitations. We collected only the completed concept maps produced by the preservice teachers and did not record the mapping process (Brandstädter et al., 2012). Therefore, we could not determine how students developed relational chains while constructing their maps, nor could we determine whether they first built an overall framework and then filled in local mechanisms, or gradually developed the map along a small number of chains. Future studies could combine drawing-trajectory recording, think-aloud protocols, and eye-tracking data to trace real-time judgments and revisions during concept-map construction (Cho et al., 2019; Stark et al., 2024), and should report more explicitly what mapping examples and strategies are demonstrated to students before mapping tasks (Jeong et al., 2025).

Manual scoring is another limitation of this study. The causal-chain completeness analysis helped identify link validity, missing intermediate mechanisms, and the continuity of mechanism chains, but these judgments still required rater interpretation. Although the inter-rater reliability reached an acceptable level, the scoring of integrative chains such as C6 still left some room for interpretation. Future research could further develop AI-supported mapping tools that combine natural language processing with graph-structural analysis to assist in identifying valid links, oversimplified links, and incorrect links, and to test the applicability of SSDA in larger samples.

Concept mapping also has boundaries when used as a single assessment instrument. A comparison between self-report assessments and scenario-based assessments of systems thinking competence has shown no significant association between what the two types of assessment capture (Davis et al., 2023). This suggests that no single instrument is likely to reflect a learner’s systems thinking in full. Future research could combine concept mapping with scenario-based tasks, interviews, or dynamic systems modeling exercises to obtain a fuller picture. One concrete direction is to examine the correlation between SSDA’s combined structural and semantic indicators and independently developed measures of systems thinking, in order to assess the extent to which this composite aligns with what other approaches capture. Such external referents would offer construct validity evidence that complements the internal structural–semantic comparison on which the present study relies.

Beyond these methodological boundaries, this study also observed that there were notable gaps in performance among the three dimensions: the representation of feedback mechanisms was generally difficult, while spatiotemporal reasoning performed better when contextual support was available. Earlier sections of the discussion provided partial explanations for this gap, but how learners transition from spatiotemporal descriptions to mechanistic explanations was not addressed in this study. How this transition unfolds cognitively, and which instructional interventions might facilitate it, are questions worth pursuing in future work.

## Data Availability

The original contributions presented in the study are included in the article/Supplementary material, further inquiries can be directed to the corresponding author.

## Supplementary material

Supplementary Appendix A Research toolkit for the concept-mapping task.

Supplementary Appendix B Semantic scoring rubric for the Structural–Semantic Dual Assessment framework.

SUPPLEMENTARY Appendix C Expert reference chains and scoring rules for the supplementary causal-chain completeness analysis.
