# Supplementary material for: Can structure speak for understanding? A dual assessment of systems thinking for sustainability in preservice STEM teachers’ concept maps
Source: Front Psychol. 2026 Jul 15;17:1846628. doi: 10.3389/fpsyg.2026.1846628 (PMC13415945; doi:10.3389/fpsyg.2026.1846628)
Supplement: Supplementary Appendix A — Research toolkit for the concept-mapping task. [file Table_1.DOCX]

**Research Toolkit for Assessing Preservice Teachers' Systems Thinking Competency**

**1. Scenario Material Overview**

Lop Nur, located in the lower reaches of the Tarim River basin, was once a vibrant saltwater lake. Historically, it served as the center of the oasis upon which the Ancient Kingdom of Loulan relied for survival. The Loulan people built canals to divert water, developed agriculture, and established the "Loulan Water Laws" to manage water resources and sustain the oasis. However, with the continuous growth of the population in the Tarim River basin and climate change, multiple rivers in the basin changed their courses and no longer injected water into Lop Nur. Consequently, the Lop Nur oasis gradually shrank, leading to the decline and eventual demise of the city. By the mid-20th century, Lop Nur, deprived of river replenishment, dried up completely, becoming known as the "Sea of Death." Meanwhile, the overall ecological condition of the Tarim River basin is not optimistic. With modern agricultural development and large-scale urban construction, upstream water resources have become increasingly scarce. Hundreds of reservoirs constructed along the Tarim River have caused a sharp reduction in downstream flow, leading to the death of vast Populus euphratica forests and further loss of ecological balance.

Potash (potassium salt) is a crucial source of agricultural fertilizer that significantly increases crop yields and is a scarce mineral resource for the country. In 1995, Chinese geologists discovered massive brine potassium deposits in the northeast direction of Lop Nur. With the development of potash mining, underground natural brine is pumped out from wells and channeled into huge artificial salt lakes for sun-drying, causing Lop Nur to recreate a landscape of "rippling blue waves."

Against the backdrop of water scarcity and ecological degradation, the Lop Nur region is currently facing dual pressures from industrial development and ecological restoration. On one hand, modern technologies are used to reinject freshwater into Lop Nur in an attempt to support ecological restoration; on the other hand, underground brine continues to be extracted to support economic development. This model of potash extraction and freshwater reinjection represents a novel resource management practice, but can it truly achieve sustainable development? This remains a question we must face.

**2. Inquiry Task: Constructing a Concept Map of "The Evolution of Lop Nur and Potash Mining"**

Please read the material above. Centering on the core issue of "What human-environment relationships are reflected in the historical evolution of Lop Nur and potash mining," attempt to draw a concept map that demonstrates your understanding of the relevant elements in the material and their relationships.

Guiding Questions: Before drawing, you may consider the following questions to help structure the basic framework of your concept map:

What are the major natural elements and human activities involved in the evolution of Lop Nur?

What interactions or influence relationships exist between these elements?

How do human activities affect water resources, the ecological environment, and regional development?

What are the contradictions and connections between potash mining and ecological restoration?

**3. Concept Map Drawing Requirements (Can be completed on paper or software):**

Step 1: Construct the Concept Network Structure

Extract at least 15 concept nodes from the material and write them in boxes (e.g., "Tarim River," "Lop Nur," "Growth in Potash Demand," "Ecological Restoration," etc.).

Use arrows to represent direct relationships between concepts, and label the relationship type above the connecting line (e.g., "Promotes," "Inhibits," "Interacts with," "Synergy," "Negative Feedback," etc.; you may define your own types).

Step 2: Label Spatiotemporal Tags

Use a red pen to add Time Tags above the concept nodes (e.g., Ancient, Modern, Future, etc.).

Use a red pen to add Space Tags below the concept nodes (e.g., Global, Local, Macro, Micro, etc.).

Label time or space information for at least 8 nodes.

Step 3: Identify System Mechanisms and Feedback Structures

List the feedback loops you identified in the diagram in the space provided below the map (Format: A → B → C → A).

Step 4: Identify Cross-Scale Relationships

After completing the drawing, review the entire map. Use a red star to highlight at least 3 cross-spatiotemporal connections (nodes connected across different time scales or spatial scales).


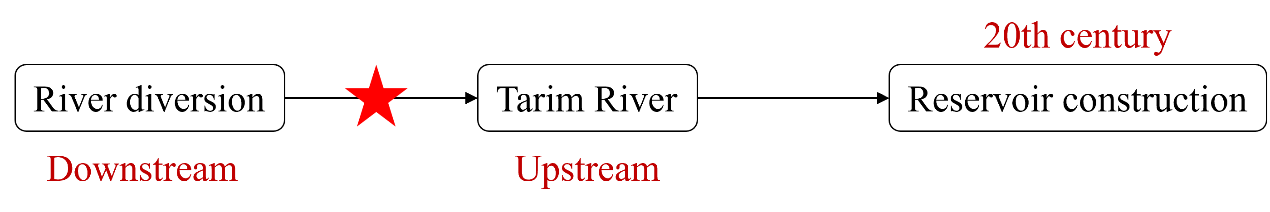


Figure A1. The illustrative example used to explain cross-spatiotemporal links
