# Supplementary material for: Can structure speak for understanding? A dual assessment of systems thinking for sustainability in preservice STEM teachers’ concept maps
Source: Front Psychol. 2026 Jul 15;17:1846628. doi: 10.3389/fpsyg.2026.1846628 (PMC13415945; doi:10.3389/fpsyg.2026.1846628)
Supplement: Supplementary Appendix B — Semantic scoring rubric for the Structural–Semantic Dual Assessment framework. [file Table_2.DOCX]

**Table B1.** Semantic Scoring Rubric for the SSDA Framework

| **Dimension** | **Score** | **Scoring Criteria** | **Example Performance** | **Key Distinction** |
| --- | --- | --- | --- | --- |
| **A: Network Structuring** | **3 (High)** | The concept map presents a highly integrated, networked system structure with clear interactions among natural, human, and social subsystems; hierarchical organization is evident and reasoning is coherent. | The map forms an interconnected network rather than a linear sequence. For example, "global potash demand" is linked to both "local water scarcity" and "ecological restoration measures," forming a driver–pressure–response model. | (Highest level) |
|  | **2 (Medium)** | Major system elements are identified and the structure is reasonably clear, but cross-system or cross-module connections are limited and overall network integration is insufficient. | Several internally coherent modules are constructed (e.g., "natural evolution," "modern development"), but connections between modules are few or oversimplified. | Separate logical modules are not yet integrated into a single, highly interconnected system network. |
|  | **1 (Low)** | Only isolated causal chains or scattered local elements are identified; relationships are simple and lack systemic integration. | "Climate → crops → yield" or "population growth → increased water use." | Scattered causal chains are not organized into logical modules with clear internal structure. |
|  | **0 (Absent)** | Only lists nouns or draws arbitrary connections with no logical relationships. | Concepts are isolated or lack directional arrows. | No valid logical or causal relationships are identified. |
| **B: Nonlinear Mechanism Deconstruction** | **3 (High)** | Feedback loops, multi-factor interactions, and nonlinear mechanisms are identified, reflecting system coupling and dynamic equilibrium. | "Vegetation loss → soil erosion → increased runoff → further vegetation loss (positive feedback)." | (Highest level) |
|  | **2 (Medium)** | Multiple causal pathways or bidirectional interactions are identified, but no complete closed loop is formed. | Both "human activity → ecological degradation" and "ecological degradation → constrains human activity" are described, but the two are not connected into a cycle. | Awareness of nonlinear thinking is present, but "cyclical" and "closed" feedback structures are not accurately identified. |
|  | **1 (Low)** | Only single linear causal relationships are identified. | "Human activity → land degradation." | Thinking is confined to unidirectional, linear causation; bidirectional interactions or multi-factor influences are not recognized. |
|  | **0 (Absent)** | No mechanistic description; only static juxtaposition of elements. | "Climate," "vegetation," and "hydrology" appear side by side. | No valid mechanisms or causal relationships are identified. |
| **C: Spatiotemporal Reasoning** | **3 (High)** | The map clearly represents the system’s temporal evolution and spatial migration/coupling, with causal arrows that span different times and spaces to establish dynamic connections. | Arrows connect the ancient "Loulan Water Laws" to modern "water resource management," showing historical continuity; or link "upstream" activities to "downstream" consequences. | (Highest level) |
|  | **2 (Medium)** | Time or space is used as an organizing framework for the concept map, but cross-spatiotemporal dynamic connections are not clearly shown. | Events are listed in "ancient–modern" order, but explicit causal arrows between different time periods are absent. | Time and space serve only as static "classification labels" or "organizing frameworks," not as causal links that convey dynamic "processes" and "coupling." |
|  | **1 (Low)** | Scattered temporal or spatial labels appear in the map, but they serve only as isolated descriptors and do not form an analytical framework. | The node "Loulan" is labeled "ancient" and "potash mining" is labeled "modern," but these labels have no temporal or spatial logical connection to other concepts. | Spatiotemporal awareness is shown (labeling), but spatiotemporal dimensions are not used to organize or analyze information. |
|  | **0 (Absent)** | The map contains no temporal or spatial information whatsoever; all relationships are purely static and decontextualized. | All relationship chains, such as "build reservoir → water reduction," lack any temporal or spatial qualification. | Spatiotemporal dimensions are entirely absent. |

Note. Each dimension is scored on a 0–3 scale. The full scoring rubric, including level descriptors and key distinctions, was used by two independent raters. Scores reported in the study are the mean of the two raters’ ratings. Since final scores are the mean of two raters' ratings, intermediate values (e.g., 2.5) may occur when raters assign adjacent scores.
