# Supplementary material for: Can structure speak for understanding? A dual assessment of systems thinking for sustainability in preservice STEM teachers’ concept maps
Source: Front Psychol. 2026 Jul 15;17:1846628. doi: 10.3389/fpsyg.2026.1846628 (PMC13415945; doi:10.3389/fpsyg.2026.1846628)
Supplement: SUPPLEMENTARY Appendix C — Expert reference chains and scoring rules for the supplementary causal-chain completeness analysis. [file Table_3.DOCX]

**Appendix C. Expert Reference Chains and Scoring Rules for the Supplementary Causal-Chain Completeness Analysis**

**C.1 Expert Reference Mechanism Chains**

Table C1 presents the six expert reference mechanism chains used in the supplementary causal-chain completeness analysis. These chains were developed from the Lop Nur task material and were used to judge whether the directed links in students’ concept maps formed valid and continuous mechanism chains. Students were not required to use exactly the same node labels as those in the expert reference chains. A link or chain was considered relevant as long as the represented concepts, causal direction, and mechanism were substantively equivalent.

**Table C1. Expert reference mechanism chains**

| **Chain ID** | **Mechanism chain** | **Expert reference chain** | **Scoring focus** |
| --- | --- | --- | --- |
| C1 | Historical hydrological change–oasis decline chain | Population growth / climate change / river diversion → reduced inflow to Lop Nur / water-resource shortage → Lop Nur oasis shrinkage / Lop Nur drying → decline of Loulan / formation of the “Sea of Death” | Whether the map represents the hydrological mechanism behind the decline of ancient Lop Nur and Loulan. A high score should show how changes in water volume led to oasis decline and further connect this process to the decline of Loulan. |
| C2 | Modern upstream development–downstream ecological degradation chain | Modern agricultural development / urban construction / reservoir construction / increased upstream water use → sharp reduction in downstream flow / water-resource shortage → Populus euphratica die-off / vegetation degradation → ecological imbalance / land desertification | Whether the map connects modern human activities, river-flow change, vegetation degradation, and ecological consequences into a mechanism chain. A high score should explain how upstream development affects downstream ecology through changes in water volume. |
| C3 | Potash development–resource production chain | Potash demand / discovery of potash resources / national demand for agricultural fertilizer → brine extraction / potash extraction → artificial salt lakes / solar evaporation process → potash production / increased agricultural yield / economic development | Whether the map shows an understanding of potash development as a resource-production activity, and whether it represents the relationship between potash production, agriculture, and economic development. |
| C4 | Potash development–ecological restoration tension chain | Potash extraction / brine extraction → water-resource disturbance / ecological pressure / restoration demand →freshwater reinjection / ecological restoration → sustainability tension between industrial development and ecological recovery | Whether the map understands “potash extraction–freshwater reinjection” as a tension between development and restoration. A high score should represent the relationships among resource extraction, ecological pressure, and restoration measures, rather than treating reinjection only as a one-directional positive governance action. |
| C5 | Ecological degradation feedback chain | Vegetation reduction / Populus euphratica die-off → intensified wind erosion / land degradation / desertification → further vegetation reduction / intensified ecological degradation | Whether the map represents a feedback relationship. A high score should show the cyclical effect through which ecological degradation further intensifies itself, rather than only describing a one-way relation such as “vegetation reduction leads to desertification.” |
| C6 | Cross-spatiotemporal water-resource governance chain | Ancient oasis water use / Loulan Water Laws / canal diversion / water-use restrictions → maintenance of ancient local oasis or basin water resources → modern upstream agricultural development / urban construction / reservoir construction / potash development → downstream flow reduction / Populus euphratica die-off / ecological degradation / Lop Nur drying → modern ecological restoration / freshwater reinjection / water-resource governance / reflection on sustainable development | Whether the map represents temporal transition, spatial-scale transmission, and governance response. A high score should not be based only on an “ancient–modern” temporal sequence or an “upstream–downstream” spatial relation. Instead, it should show whether students organize human water use or governance activities across different periods, hydrological–ecological impacts across spatial scales, and subsequent governance responses into a directed mechanism chain. |

**C.2 Scoring Rules**

Each expert reference chain was scored on a 0–3 scale. The scoring was based on the directed links actually represented in the concept map. When relationship labels were provided, raters used them to interpret the meaning of the links. When relationship labels were absent, raters mainly judged whether directed links were present, whether the causal direction was reasonable, and whether the links formed a continuous mechanism chain.

**Table C2. Scoring rules for each expert reference mechanism chain**

| **Score** | **Scoring criterion** |
| --- | --- |
| 3 | The concept map forms a complete cause–intermediate process–outcome chain. The causal direction is correct, the relations are reasonable, and the main mechanism represented by the expert chain is continuously presented. |
| 2 | The main mechanism chain is present, but one important intermediate link, downstream consequence, or governance response is missing. The represented chain remains generally interpretable. |
| 1 | The concept map presents only one valid direct relation, or expresses the general direction of the mechanism in a broad way, but does not form a continuous mechanism chain. |
| 0 | The concept map does not present the relevant mechanism chain, or the causal direction is incorrect, or the represented relation cannot be supported by the task material. |

The scoring allowed 0.5-point intervals to distinguish intermediate performance between adjacent scoring anchors. When a concept map contained a distal causal link such as A→C or A→D, and the broad direction was plausible but key intermediate mechanisms were omitted, a score of 0.5 could be assigned. When the map contained more than one locally valid relation and therefore showed stronger evidence than a single direct relation, but still did not form a continuous mechanism chain, a score of 1.5 could be assigned. When the main mechanism was relatively clear but still missed one important intermediate process, downstream outcome, or governance response, a score of 2.5 could be assigned. Thus, distal links could receive partial credit, but they were treated as complete mechanism chains only when the relevant intermediate and downstream relations were also represented with sufficient completeness.

**C.3 Annotated Example of a Problematic Structure**

Figure C1 provides an annotated example from participant S28. The red elements in the map are the participant’s original temporal labels, spatial labels, and cross-spatiotemporal markings. The blue dashed box and accompanying blue text were added by the researchers and were not part of the participant’s original concept map.


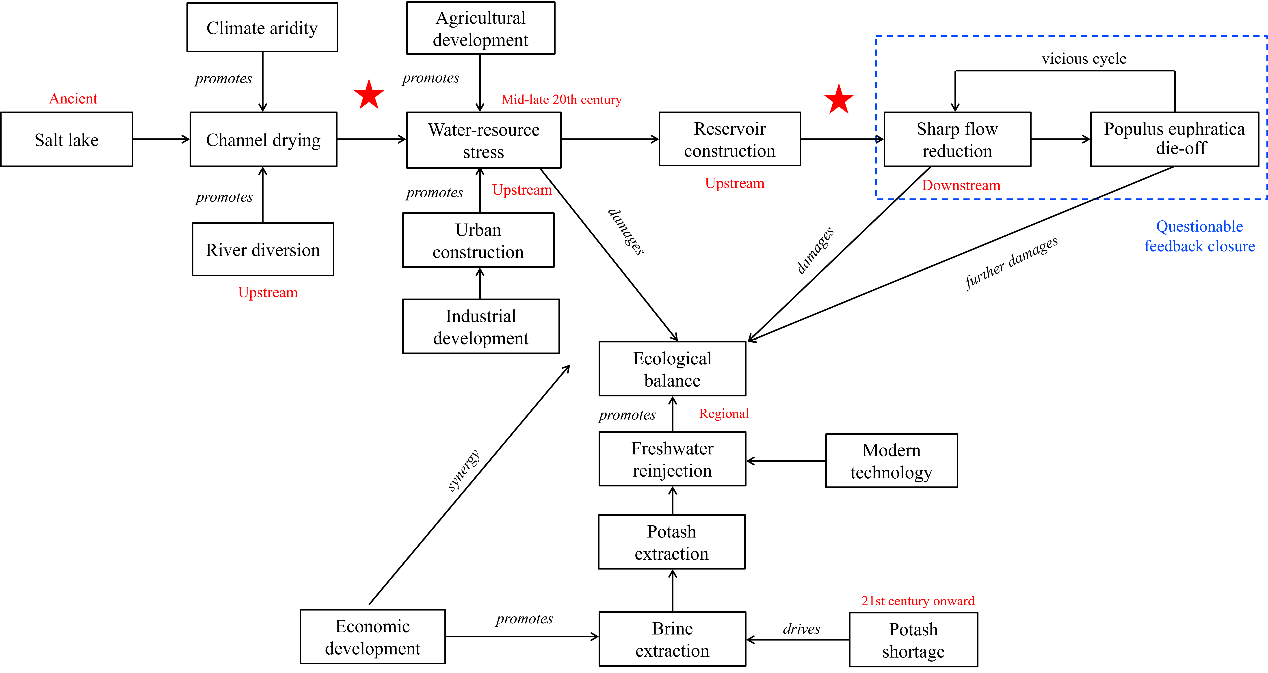


**Figure C1. Annotated example of a questionable feedback structure in S28.**

The blue dashed box marks the feedback closure between “Sharp flow reduction” and “Populus euphratica die-off.” In the map, the direction from “Sharp flow reduction” to “Populus euphratica die-off” corresponds to the mechanism in the task material, in which reduced downstream flow leads to the death of Populus euphratica forests. However, the student further linked “Populus euphratica die-off” back to “Sharp flow reduction” without explaining through what intermediate mechanism this reverse effect would occur. If this feedback was intended to suggest that vegetation loss weakens water conservation capacity, alters local microclimate, or affects groundwater conditions, its object of influence would be closer to local moisture conditions. By contrast, the “Sharp flow reduction” node in the map mainly refers to changes in downstream river flow, which are more strongly shaped by basin-scale processes such as upstream water use and reservoir construction. Therefore, this closed structure involved scale misalignment and a conflation of primary and secondary mechanisms. In the scoring process, it was treated as a questionable feedback closure rather than a complete and valid mechanism chain.
